# Statistically downscaled CMIP6 ocean variables for European waters

**DOI:** 10.1038/s41598-024-51160-1

**Published:** 2024-01-12

**Authors:** Trond Kristiansen, Momme Butenschön, Myron A. Peck

**Affiliations:** 1 Farallon Institute, Petaluma, CA USA; 2 CMCC Foundation—Euro-Mediterranean Center on Climate Change, Bologna, Italy; 3 Department of Coastal Systems, Royal Netherlands Institute for Sea Research, Texel, The Netherlands; 4 Actea Inc, San Francisco, CA USA; 5 Norwegian Institute for Water Research, Oslo, Norway

**Keywords:** Climate sciences, Environmental sciences, Ocean sciences, Marine biology, Physical oceanography

## Abstract

Climate change impact studies need climate projections for different scenarios and at scales relevant to planning and management, preferably for a variety of models and realizations to capture the uncertainty in these models. To address current gaps, we statistically downscaled (SD) 3–7 CMIP6 models for five key indicators of marine habitat conditions: temperature, salinity, pH, oxygen, and chlorophyll across European waters for three climate scenarios SSP1-2.6, SSP2-4.5, and SSP5-8.5. Results provide ensemble averages and uncertainty estimates that can serve as input data for projecting the potential success of a range of Nature-based Solutions, including the restoration of habitat-forming species such as seagrass in the Mediterranean and kelp in coastal areas of Portugal and Norway. Evaluation of the ensemble with observations from four European regions (North Sea, Baltic Sea, Bay of Biscay, and Mediterranean Sea) indicates that the SD projections realistically capture the climatological conditions of the historical period 1993–2020. Model skill (Liu-mean efficiency, Pearson correlation) clearly improves for both surface temperature and oxygen across all regions with respect to the original ESMs demonstrating a higher skill for temperature compared to oxygen. Warming is evident across all areas and large differences among scenarios fully emerge from the background uncertainties related to internal variability and model differences in the second half of the century. Scenario-specific differences in acidification significantly emerge from model uncertainty and internal variability leading to distinct trajectories in surface pH starting before mid-century (in some cases starting from present day). Deoxygenation is also present across all domains, but the climate signal was significantly weaker compared to the other two indicators when compared to model uncertainty and internal variability, and the impact of different greenhouse gas trajectories is less distinct. The substantial regional and local heterogeneity in these three abiotic indicators underscores the need for highly spatially resolved physical and biogeochemical projections to understand how climate change may impact marine ecosystems.

## Introduction

Climate change affects the physics and biology of marine ecosystems through warming, acidification, deoxygenation, and changes in productivity (e.g., chlorophyll concentration). Ocean observations suggest that these changes have taken place at a more rapid pace than previously expected^1^ and can manifest in a range of ways. For example, there is evidence of increased frequencies of extreme heatwaves in the Pacific Ocean^2^ and expansion of deoxygenated zones, which can be attributed to eutrophication^3,4^, warming (decreased oxygen saturation), and stratification (decreased mixing and ventilation)^5^. Combined stressor impacts can change species distribution, phenology, survival, and growth, as well as habitat and spawning conditions. Effective decision-making on how to mitigate or adapt to climate change requires detailed data on future ocean temperatures, acidification, changes in oxygen, and ocean productivity.

Scientific analysis and conclusions featured in the sixth assessment report of the IPCC are based largely on the critically important output from the Coupled Model Intercomparison Project (CMIP6) and historical observations. CMIP6 uses Earth System Models (ESMs) or General Circulation Models (GCMs) that can simulate the various physical, biological, and chemical processes within the atmosphere, ocean, ice, and land and how those processes combine to affect the global climate. These models provide possible climate trajectories for the future based on a range of greenhouse gas concentration scenarios and Shared Socioeconomic Pathways (SSP), which provide a wealth of information at large spatial scales^6^. Still, working with CMIP6 model outputs can be technically challenging^7^. For example, the grid structure of CMIP6 models can be complex with non-uniform representation of longitude and latitude grid points, which are used to avoid singularities at the poles and to enhance resolution closer to the equator. In addition, a single model variable may be split into hundreds of files that need to be concatenated in time. Modelled variables may also be biased compared to observations. Working with these global datasets presents several logistical challenges. They require large storage space and cannot be sub-sampled prior to downloading from the original data archives, although both Google Cloud and Amazon Web Services now provide a subset of the model data improving their accessibility. CMIP6 associated terminology can also be impenetrable to non-experts^7^. As a result, the outputs of CMIP6 across a range of spatial and temporal scales often prohibit the use by non-experts, limiting their use for further ecosystem impact analysis.

The coarse resolution of the CMIP6 models (most models represent the ocean at roughly 1$^\circ$ × 1$^\circ$ resolution in longitude and latitude grid) does not resolve mesoscale features (e.g., eddies) essential to understanding dynamical processes in coastal regions. Higher resolution climate projections for coastal and shelf areas are increasingly needed as inputs to adaptation and mitigation planning as well as management of marine resources^8^. Downscaling global climate projections to a higher resolution can preserve the large-scale climate signal while capturing local variability and dynamics^9^. While several dynamical downscaling products exist for regional ocean domains^10,11^, these products lack the conceptual and standardized approach of the CMIP experiments, or the Coordinated REgional Downscaling Experiment (CORDEX) program for regional atmospheric models. Dynamically downscaled products often focus on a single or limited set of climate models and scenarios^12,13^. The lack of consistent ensembles with diversity in models and scenarios strongly limits the comparability of results across different systems and does not adequately quantify the uncertainty in the physical and biogeochemical projections^8^, which is critical for risk assessment, ecosystem-based management, and informing mitigation and adaptation policies.

**Figure 1:**
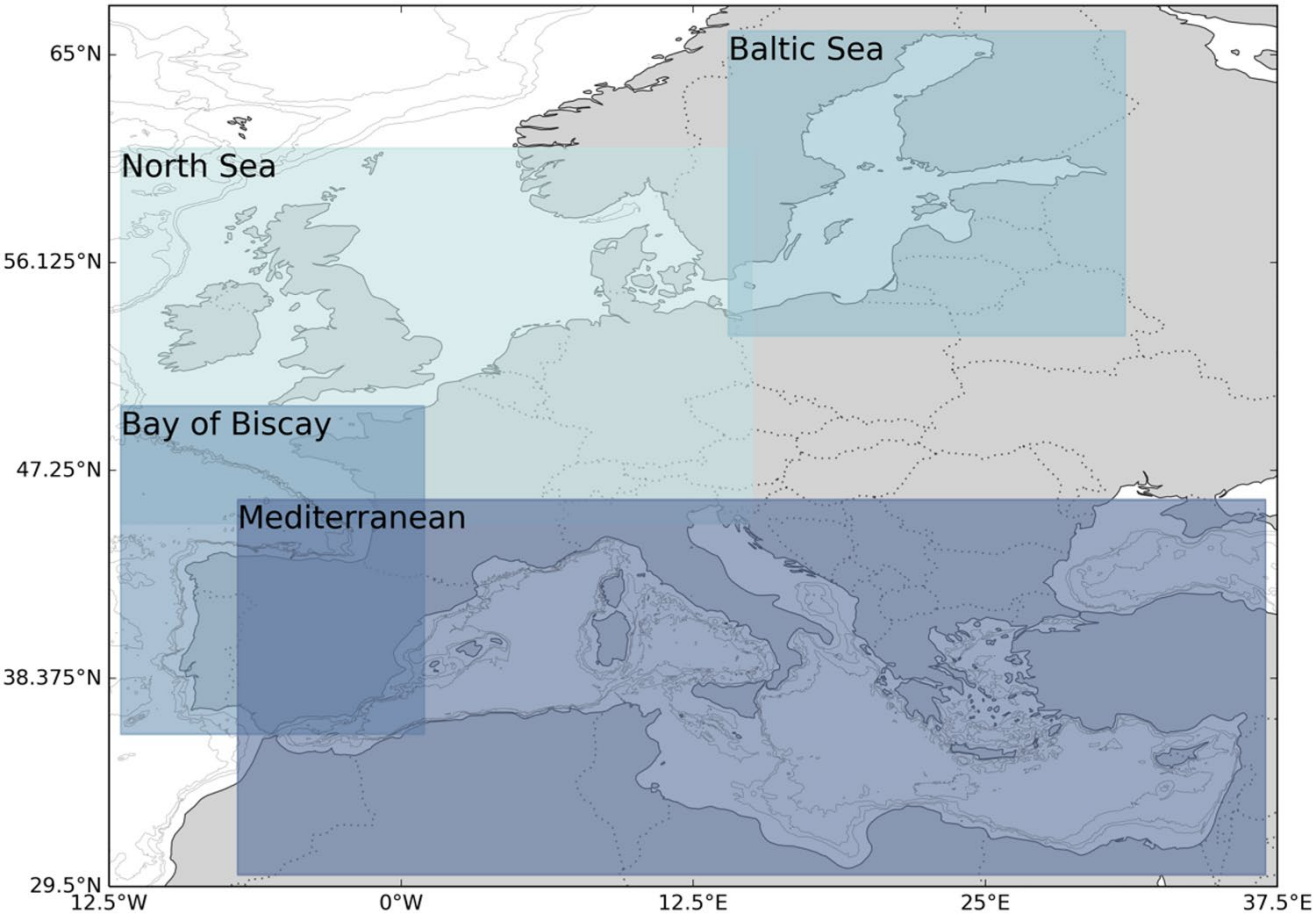
Map showing the four European focus regions of FutureMARES where statistical downscaling of CMIP6 projections was applied: the Baltic Sea, the North Sea, the Bay of Biscay, and the Mediterranean Sea. The Black Sea is not included in the Mediterranean region.

The EU-funded research project FutureMARES aims to provide socially and economically viable actions and strategies that support Nature-based Solutions (NBS) for climate change adaptation and mitigation across Europe. To meet these ambitious goals, the datasets developed here were explicitly designed to deliver consistent climate-driven projections of change in physical and biogeochemical factors at the spatial scales relevant for planning and management across European regional seas. To do this, we bias-corrected and statistically downscaled individual CMIP6 models to provide an ensemble of high-resolution climate projections for different IPCC scenarios. Here, we present the methodology, evaluation, and uncertainty analysis of the downscaled dataset products made publicly available through Zenodo (zenodo.org). The ensemble high-resolution climate dataset provides projections for 1993–2100 (monthly) at roughly 8 km (1/12th degree) resolution for four European regions: the North Sea, the Baltic Sea, the Bay of Biscay, and the Mediterranean Sea (Fig. 1).

## Methods

The methodology applied to statistically bias-correct and downscale large-scale CMIP6 climate projections to the regional level was conducted in four steps: (1) preparing the input data, (2) bias-correcting with respect to observations, (3) statistically downscaling the bias-corrected fields to higher resolution, and (4) creating ensemble statistics of the downscaled models.

### Preparation of global climate data

The raw data from global climate models (GCM) and Earth System Models (ESMs) can be represented on various global ocean grids. Some of these grids have higher resolution in one part of the world, e.g., around the equator, while others can have three poles to avoid the singularity in the ocean at 90° N. To be able to work consistently with these model outputs, we interpolated the data to a uniform cartesian grid of 0.5° × 0.5° longitude-latitude. We employed the Earth System Modelling Framework (ESMF)^14^ to allow fast interpolation within a tested framework. We also use the Python xesmf interface^15^ to the ESMF package, which further simplified the conversion from the native to a uniform grid. We used the xMIP package for pre-processing the CMIP6 data^16^. Once the GCM/ESM data were converted to a standard grid, we performed a bias-corrected statistical downscaling of the data, a two-step process: (1) bias-correction and (2) statistical downscaling.

### Bias-correction

The GCMs and ESMs within CMIP6 were designed to represent the probability distributions, variability, and observed trends in physical and biological variables and not to exactly replicate individual features in time and space as reanalysis systems would do for the past or forecasting systems for the near future. For this reason, GCMs and ESMs are inherently biased from historical observations. To correct the offset in the global models, we performed a bias-correction where the large-scale climate signal was constrained to observed values of the range and variability using detrended quantile mapping (DQM) transformations^17,18^. The DQM is designed to remove biases across all quantiles, effectively aligning the data distribution of the model data relative to the observed values^18^. Here, we use the ocean reanalysis GLORYS12V1^10^ as observations to quantify the biases present in the GCM/ESMs. The DQM method was trained with the historical (1993–2020) GLORYS12V1 reanalysis and applied to the historical GCM/ESM data to calculate the transform function which was used to adjust the detrended quantiles for the future projections (2020–2100). Once the timeseries had been adjusted using the DQM methodology, the trend was added back to the timeseries^9^. This approach ensures that the trend from the GCMs and ESMs is preserved in the downscaled product. The bias-correction was performed at the resolution of the interpolated GCM or ESM, which is 0.5° × 0.5° latitude-longitude. The global ocean physics of the GLORYS12V1 reanalysis at 1/12th degrees resolution have been thoroughly validated against observations^10,19^. The GLORYS12V1 reanalysis assimilates available historical data (e.g., satellite, CTD, XBT, buoys) for 1993-01-01 to 2019-12-31 and represents state-of-the-art hydrodynamic modeling. GLORYS12V1^19^ is developed by Mercator Ocean and is an operational service from the Copernicus Marine Service Center (marine.copernicus.eu).

Historical biogeochemical data such as oxygen, chlorophyll, and pH were obtained from the biological model Global Ocean Biogeochemistry hindcast (GOBH) from Mercator Ocean distributed via the Copernicus Marine Service. The GOBH model^20^ uses the PISCES model to represent biogeochemistry and physics from the FREEGLORYS2V4 model, which is a non-assimilative version of the GLORYS2V4 reanalysis model. The GOBH and FREEGLORYS2V4 models were run at a resolution of 1/4th degree. To align the downscaled physical and biogeochemical results directly, we interpolated (bilinear) and extrapolated these model data onto the physical model grid, allowing the final biological downscaled data to be at 1/12th degree resolution. As the bathymetry along the coastline of the biological model is coarser than the physical model, we extrapolated to the destination point by using the weighted average of the eight nearest source points. The weight is the reciprocal of the distance of the source point from the destination point raised to a power 2 (the inverse weighted distance method)^14^. The documentation for GOBH states that the model holds a global bias in pH of 0.02, making it slightly more acidic compared to observations. The model is able to reproduce observed surface and sub-surface oxygen concentrations including the oxygen minimum zone^20^. However, being a hindcast, as all dynamic ocean models, this dataset contains some biases with respect to the real world which will be inherited by the downscaled products.

### Statistical downscaling

The statistical downscaling (SD) allowed us to establish an empirical relationship between high-resolution historical and large-scale climate indicators and apply these statistics to produce local climate projections. The bias-corrected fields at 0.5° × 0.5° longitude-latitude resolution were used as input to the DQM statistical downscaling algorithm together with the high-resolution GLORYS12V1 reanalysis to provide ESM sub-grid variability. This involved (1) determine a scaling factor that allow the mean of the historical bias-corrected CMIP6 projection to be equal to the mean of the historical GLORYS12V1/GOBH timeseries, (2) remove the trend from both timeseries (3) calculate the adjustment factors between the quantiles of the two timeseries, (4) apply the scaling factor to the future projections, (5) match the quantiles of the detrended projections and apply the adjustment factor, and (6) add back the trend to the projections^18^. The GLORYS12V1 and GOBH models have 50 and 75 vertical depth levels, respectively, which were linearly interpolated if the bias correction and downscaling were performed at an intermediate depth level. Linear interpolation was also performed on the global climate model outputs as downscaling was done at individual, fixed depth levels (e.g., 5 m, 25 m). The exception was the bottom depth, where each grid point had a unique depth level, and the ESMs were interpolated to the GLORYS bathymetry.

### Ensemble product

This downscaling was performed for a range of CMIP6 models (3 to 7) per variable per climate scenario (see Table 1 for an overview), and the final product to be used by researchers was the ensemble of these individual downscaled models. The ensemble provides datasets for five indicators of marine habitat conditions (temperature (°C), salinity, pH, dissolved oxygen (ml/l), chlorophyll (kg/m^3^)). These data were provided at three distinct depth levels (surface (5 m), sub-surface (25 m), and seafloor) for 1993–2100 under three different future scenarios (SSP1-2.6, SSP2-4.5, and SSP5-8.5). Within the datasets, the ensemble mean is provided along with standard deviations and 2.5, 50, and 97.5 percentiles, depicting the spread of the ensemble at each point in space and time. Although some of the CMIP6 models were downscaled for multiple model realizations (Table 1), each downscaled realization held equal weight when calculating the ensemble product which may favour these models results compared to single variant models, a weakness that can potentially be addressed by ensemble weighting based on model independence and performance^21^. This approach will be attempted in a future iteration of this dataset. The downscaled results were stored as compressed NetCDF4 files containing self-describing metadata of the downscaled variable.

**Table 1:** Climate scenarios, realizations, and variables downscaled for each CMIP6 model used to create the ensembles.

Model name	Realization	SSP1-2.6, SSP2-4.5, and SSP5-8.5														
		O_2_			Temperature			Chlorophyll			pH			Salinity		
IPSL-CM6A-LR (Boucher et al., 2020)	r1i1p1f1	x	x	x	x	x	x				x	x	x	x	x	x
	r3i1p1f1	x	x	x	x	x	x				x	x	x	x	x	x
MPI-ESM1-2-LR (Mauritsen et al., 2019)	r1i1p1f1	x	x	x	x		x	x	x	x	x	x	x	x	x	x
	r2i1p1f1	x	x	x	x	x	x	x	x	x	x	x	x	x	x	x
GFDL-ESM4 (Dunne et al., 2020)	r1i1p1f1				x	x	x							x	x	x
CMCC-ESM2 (Lovato et al., 2022)	r1i1p1f1	x	x	x	x	x	x	x	x	x	x	x	x	x	x	x
CMCC-CM2-SR5 (Cherchi et al., 2018)	r1i1p1f1				x	x	x							x	x	x

### Climate scenarios

Global climate models are complex tools that allow researchers to explore how combinations of stressors interact and affect the Earths’ climate system. These models use global greenhouse gas concentrations emerging for the radiative forcing targets of the Representative Concentration Pathways—RCPs, under different shared socioeconomic pathways (SSPs) up to 2100 according to the ScenarioMIP protocol^22^. For the sixth Intergovernmental Panel on Climate Change (IPCC) report, five narratives provided alternative socio-economic developments for the world, including sustainable development (SSP1), regional rivalry (SSP3), regional inequality (SSP4), fossil-fuelled development (SSP5), and middle-of-the-road development (SSP2). While, in principle, the two development streams of climate and socio-economic scenarios are independent, some combinations are more likely than others. This dataset focuses on the combinations SSP1-RCP2.6, SSP2-RCP4.5, and SSP5-RCP8.5 which are part of the ScenarioMIP Tier 1 simulations and available across various ESMs. The selection of CMIP6 models and variants was made based on each model's overall performance and skill^23^, and model availability across variables and scenarios.

### Evaluation of ensemble

Several techniques and datasets were applied to validate the ensemble downscaled climate projections. This involved comparing the ensemble results with comprehensive observational datasets, focusing on temperature, and oxygen as key variables. First, we compared the ensemble and its members against the spatially continuous World Ocean Atlas (WOA) climatology for surface temperature (WOA23, 1/4° degree resolution) and dissolved oxygen (WOA18, 1° degree resolution) to obtain a spatially continuous gapless comparison^24^. The World Ocean Atlas is a collection of objectively analysed profile data (e.g., temperature, oxygen) from the World Ocean Database. The performance of the downscaled product with respect to the original GCMs and ESMs was assessed using several standard metrics. Each metric was computed for the spatial fields of the seasonal climatology of surface temperature and surface dissolved oxygen. The seasonal climatologies were compared against the WOA climatology and the metrics averaged over seasons. These datasets come at a resolution that is comparable to the original ESM data and allows us to compare the SD with the raw ESM outputs and identify the improvements of the downscaled product beyond the added value of increased resolution.

The metrics calculated and compared were:

- the ratio of the model mean over the mean of observations (α) which assessed the overall bias of the model fields for each season;
- the ratio of the model standard deviation over the standard deviation of observations (β) which assessed the overall spread of the model fields for each season;
- the Pearson correlation coefficient (*ρ*) of the spatial fields from the model and from the observations which estimated the spatial mismatch of local features and bias.

These three metrics were also combined into a summary metric providing a single number for model skill. Several approaches have been previously used to report model skill^25–27^. We chose the Liu-mean efficiency skill score (LSE), which provides a more balanced representation of the individual components that have been shown to yield superior results to the previous methods when used in model optimizations^27^. This skill score combines the individual metrics according to:

$$LSE = 1 - \sqrt {\left( {\rho \alpha - 1} \right)^{2} + \left( {\beta - 1} \right)^{2} } ,$$

A Liu skill score of 1 represents a perfect comparison with observations, while values below 1 indicate a diminishing level of comparison between ensemble and observations. Specifically, the first component of the skill score combining the correlation coefficient and the ratio of the standard deviations evaluates the distance of the linear regression slope between the ensemble dataset and the reference observations to 1. The second component is the non-dimensional measure of the overall bias of the two datasets. To illustrate the numerical values of the skill score, here are a few examples: a completely uncorrelated relationship of the two datasets would yield 0 for the first component of the skill score leading to negative values of the skill score for any further deficiency in the second component. Similarly, if the standard deviations or the mean of the ensemble would reach twice that of the observations, the skill would become 0 or smaller even for perfect correlation between model and observations.

In addition, to best validate surface and bottom values of temperature, and oxygen, we compared the ensemble data with in-situ observations obtained from a variety of platforms (e.g., buoys, profiles, and shipboard CTD, pump data, mooring data,) available from the ICES online database (www.ices.dk) that has extensive coverage of North Sea, Baltic Sea and the North-Eastern Atlantic. For the Mediterranean Sea we used the GLODAP^28,29^ database instead of the ICES database as it provides better coverage of the basin. The comparisons between ensemble values and observations from either ICES or GLODAP were done by identifying observations located within 100 m horizontal distance from any downscaled grid locations and ± 1 m vertically up or down from the depth of the ensemble grid location. This allowed us to compare the nearest observations in space (latitude, longitude, and depth) to the downscaled data point, while also being agnostic with respect to seasonality. Hence we did not compare the timestamp of ensemble versus observation but rather collected all data within the 27-year time period to assess overall distributions. The ensemble and observed distributions of the range in values with depth of both temperature and oxygen for each of the four regions for the period 1993–2020 were quantified.

### Uncertainty assessment

The uncertainties in the downscaled ensemble product were assessed by evaluating the three principal categories of uncertainty in these types of simulations^30^.

- Scenario uncertainty is the uncertainty related to the different greenhouse gas concentrations and shared socioeconomic pathways affecting the global climate;
- Model uncertainty, the uncertainty related to the different structures, and parametrization of the GCMs and ESMs;
- Internal variability, the uncertainty related to the natural variability of the climate system in the absence of external forcing caused by intrinsic processes of the ocean, atmosphere, and land.

These three components have different relative importance at different lead times of a climate projection, with the latter two generally dominating at shorter time scales. The scenario uncertainty tends to become increasingly important as the projection evolves with time due to the increasing spread in greenhouse gas forcing among the scenario pathways^31^.

Our uncertainty assessment illustrated the spatial distribution of changes in three key ecosystem indicators induced by anthropogenic greenhouse gas emissions, relating them to each source of uncertainty via their ratio (change/uncertainty). Changes are considered significant where the ratio exceeds 1.

In our assessment, future changes were computed from the ensemble mean for the middle of the road Scenario (SSP2-4.5) for the mid- and long-term IPCC assessment periods (2041–2060 and 2081–2100, respectively) by subtracting the mean conditions of the present-day time slice (1995–2014) from the mean conditions of the future time slice.

The uncertainty fields used to compute the significance ratios for each source of uncertainty were computed as follows:

- Scenario uncertainty: changes were computed for each scenario as for the baseline scenario SSP2-4.5 described above. The uncertainty was then determined as the min–max range of all scenarios in each spatial pixel.
- Model uncertainty: changes were computed for each individual model realization of the baseline scenario SSP2-4.5 as for the ensemble mean described above. The uncertainty was then determined as the min–max range of all model realizations in each spatial pixel.
- Internal variability was estimated by the difference between the maximum and minimum value of the annual mean time series of the ensemble mean of the baseline scenario projection with long-term trends removed, which was achieved by applying a running average filter with a 21-year window over the original time series.

The results from the uncertainty assessment were provided as spatial maps of the change in each variable and its magnitude relative to each source of uncertainty. This spatial representation illustrates the differences in importance of the three sources of uncertainty at different locations.

## Results

### Evaluation

The statistical downscaling improved model skill for oxygen and temperature when comparing SD products and the original ESMs (Tables 2, 3; see Tables S1–S10 for more details) across all regions. Temperature shows a higher skill than oxygen, with a difference of around 0.2–0.4 points between the ESM Liu-mean efficiency skill score and the SD. It is worth noting that the SD significantly reduced model differences in performance, while for the original ESMs, the inter-model differences can be substantial.

**Table 2:**
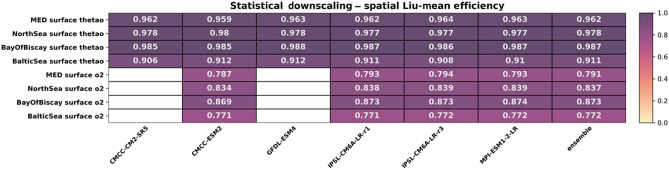
Liu-mean efficiency of the statistical downscaling products for surface temperature (thetao (°C)) and surface oxygen (O_2_ (ml/l)) for each basin.

The evaluation is based on present-day seasonal averages against WOA climatology.

**Table 3:**
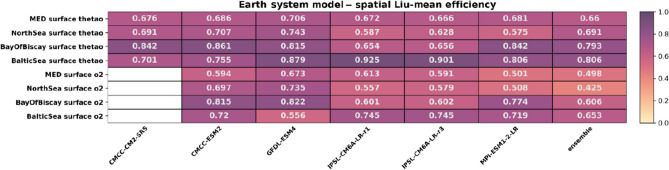
Liu-mean efficiency of the original earth system models for surface temperature (thetao (°C)) and surface oxygen (O_2_ (ml/l)) for each basin.

The evaluation is based on present-day seasonal averages against WOA climatology.

The decomposition of the performance analysis into its components (Tables S1–S10) suggests that, for the SD products, reduced model skill was attributable to the spatial correlation coefficient (Table S1–S2), while the ratio of means and ratio of standard deviations showed close to perfect matches for these products (Table S3–S5). For the original GCM and ESM simulations, the ratio of means was also generally very close to 1 (Tables S9, S10). Still, both the Pearson correlation and the ratio of standard deviation indicate substantial shortcomings in skill (Table S6, S9). For the SD products, the main driver for lack of skill was a weak correlation which is significantly stronger than the mismatches of standard deviations.

**Figure 2:**
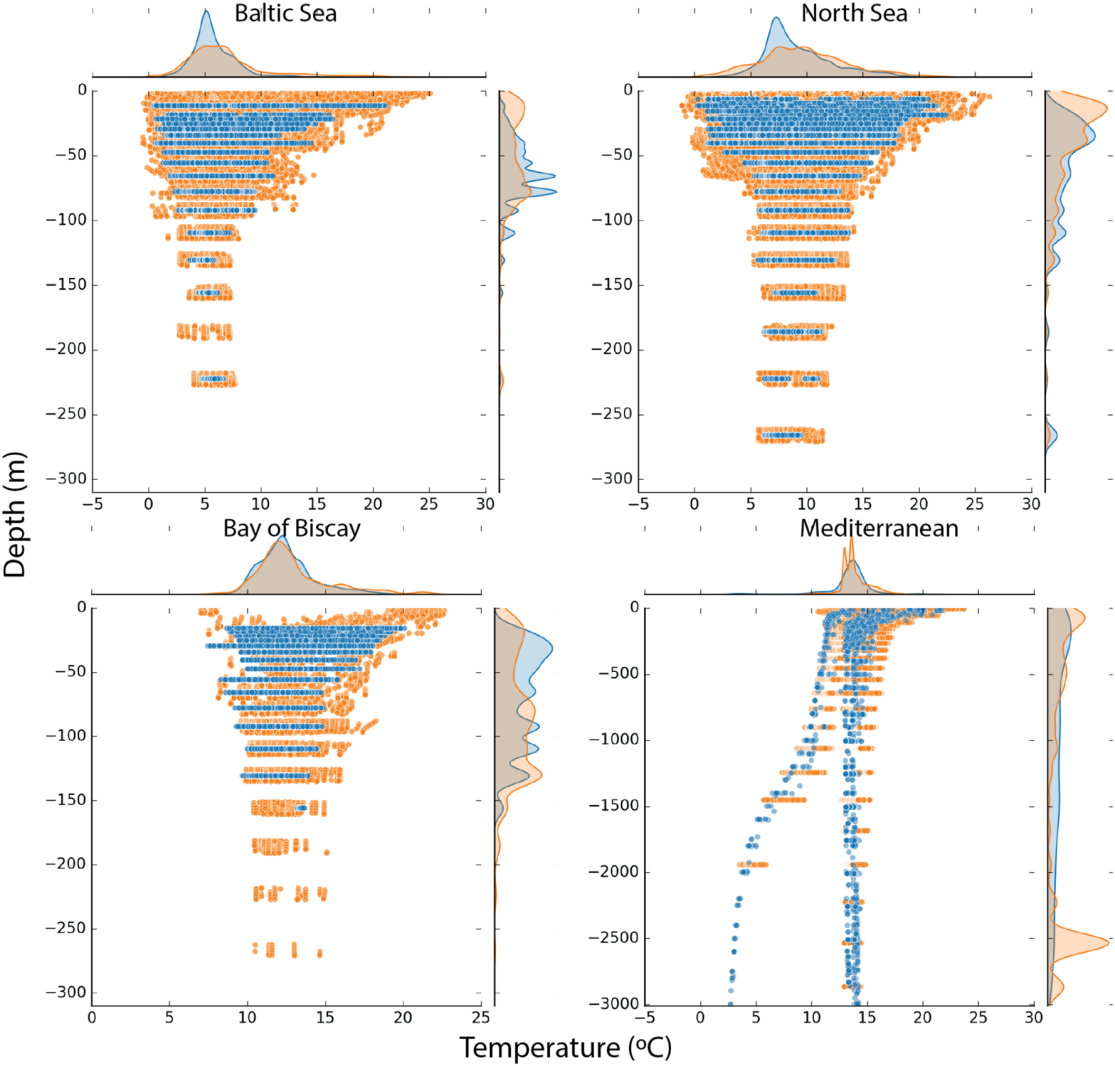
Comparison between the ensemble (blue) downscaled bottom temperature (thetao (°C)) and observations (orange) extracted from the ICES (www.ices.dk) and GLODAP databases^29^. For the Baltic Sea (upper left), the North Sea (upper right), and the Bay of Biscay (lower left) we used data from ICES for the comparison, while for the Mediterranean Sea we used GLODAP^28^. The comparison used all available data for the period 1993–2020. The frequency diagrams indicate the overlap in distributional value range (top) and depth (left side) between the observations and the downscaled data.

**Figure 3:**
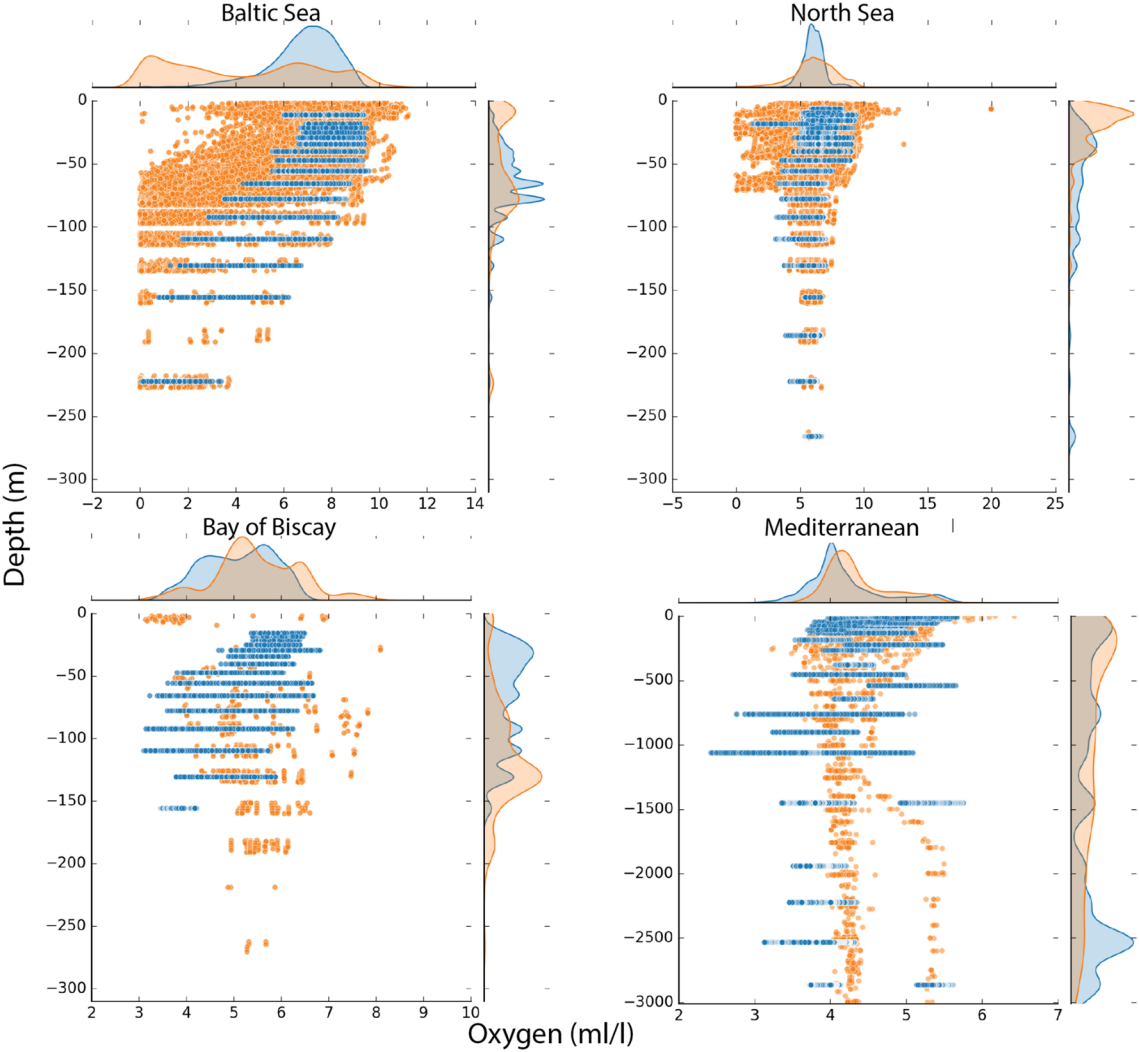
Comparison between the ensemble (blue) downscaled bottom oxygen (O_2_ (ml/l)) and shipboard observations (orange) extracted from the ICES (www.ices.dk) and the GLODAP databases^28^. For the Baltic Sea (upper left), the North Sea (upper right), and the Bay of Biscay (lower left) we used data from ICES for the comparison, while for the Mediterranean Sea we used GLODAP. The comparison uses all available data for the period 1993–2020. The frequency diagrams indicate the overlap in distributional value range (top) and depth (left side) between the observations and the downscaled data.

The relatively coarse resolution of the WOA data, particularly oxygen, is a challenge for representing enclosed regions such as the Baltic Sea. For that reason, the ensemble downscaled data for bottom temperature (Fig. 2) and oxygen (Fig. 3) were compared with observations from the ICES database for the Baltic Sea, the Bay of Biscay and for the North Sea, while data for the Mediterranean were compared against the GLODAP database. In total, we extracted a large number of observations totaling 39,797 data points for the Baltic Sea, 126,401 for the North Sea, 9,385 for the Bay of Biscay, and 6,306 for the Mediterranean (Fig. S1). The depth and range distributions of bottom temperature (Fig. 2) are comparable to the observations for all regions. For the Mediterranean, the bifurcation between the Eastern and Western Mediterranean basins can be identified in both the temperature and oxygen data. When compared with observations, the downscaled temperature data realistically captured the value range as a function of depth (Fig. 2) for all regions. The oxygen data in the ensemble exhibited a narrower range across all depths compared to the observed values in the Baltic and North Sea (Fig. 3). Conversely, in the Bay of Biscay, the available oxygen observations were limited and dispersed over a wider area, making direct comparison with the ensemble data more challenging. In the Baltic Sea, the ensemble data show higher oxygen concentrations than the observations, especially in the waters shallower than 100 m (Fig. 3). This difference is also seen in the upper 100 m in the North Sea where values below 1.4 ml/L is typically classified as hypoxic conditions can be found in the observations (Fig. 3). The bottom pH from the ensemble products were compared with the ICES and GLODAP databases but very few observations matched our filtering criteria, although the ones that did compared reasonably well (Fig. S2).

### Uncertainty across regions

In this section, we illustrate the changes induced by anthropogenic greenhouse gas emissions for the three variables previously evaluated, sea surface temperature representing warming, surface pH representing acidification, and bottom dissolved oxygen representing deoxygenation. The significance of the induced changes was further analysed by comparing them to three separate sources of uncertainty in climate projections: (1) internal variability, (2) model uncertainty, and (3) scenario uncertainty. In the following subsections, we present the results of this analysis by region.

#### Mediterranean Sea

**Figure 4:**
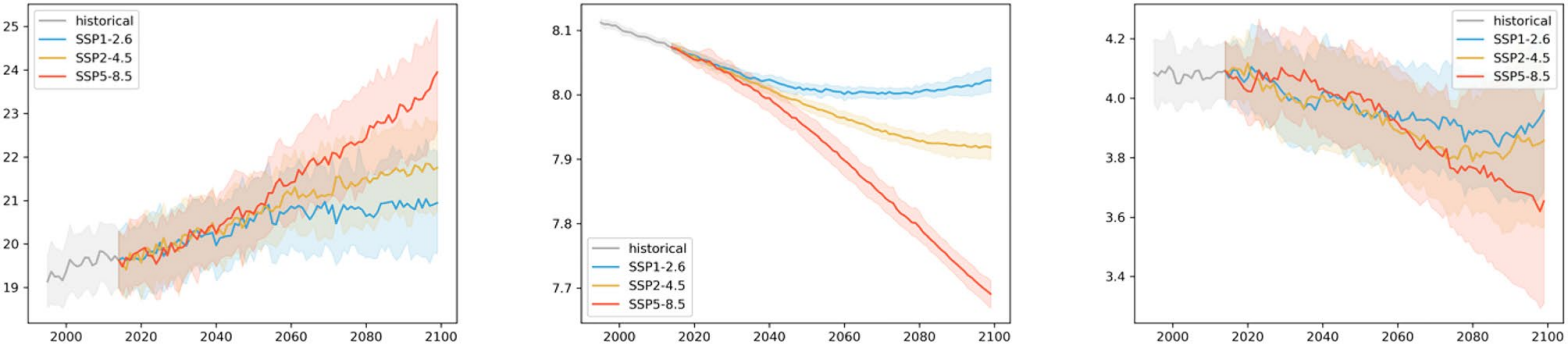
Time series of Mediterranean Sea average surface temperature (°C), pH, and bottom oxygen (ml/l) over the historical time period and the three scenarios. Solid lines show the ensemble, and shaded areas show the ensemble spread based on the 2.5 and 97.5 percentiles of the model distributions.

Figure 4 shows the Mediterranean Sea basin average time series of the three ecosystem indicators from 1993 up to the end of this century for the three scenarios represented by the SD ensemble product. The patterns are qualitatively comparable to those observed for the global mean trajectories^32,33^. For the no-mitigation scenario SSP5-8.5, the bulk surface temperature of the Mediterranean Sea gradually increases to 5 °C higher than the present day. For the middle of the road scenario SSP2-4.5, the increase in temperature from present-day is lower, reaching approximately 2 °C. For the strongly mitigated scenario SSP1-2.6, the temperature initially increases and then stabilizes towards the middle of the century at around 1.5 °C of warming. The model spread is moderately high (2.5 to 3.0 °C), so the differences between the two scenarios producing weaker warming partially overlap. In contrast, for the high emissions scenario (SSP5-8.5), there is stronger warming and the difference in temperature emerges from the model uncertainty. Interannual variability is low compared to long-term changes. Regarding ocean acidification, in SSP5-8.5 a strong, gradual decrease in ocean pH occurs to about 0.4 units from present-day conditions, while the decline in SSP2-4.5 is less marked, and pH stabilizes by the end of the century in SSP2-4.5, after a 0.15 unit decrease. The SSP1-2.6 scenario suggests a slightly reversing trend, limiting the overall decrease in pH to less than 0.1 units. Uncertainty for this pressure is inherently low, and differences in the changes among the scenarios are clear. For bottom oxygen, uncertainty is highest relative to the changes observed among the three scenarios. For all three scenarios, oxygen decreases by approximately 0.5, 0.2, and 0.1 ml/l for SSP5-8.5, SSP2-4.5, and SSP1-2.6, respectively.

**Figure 5:**
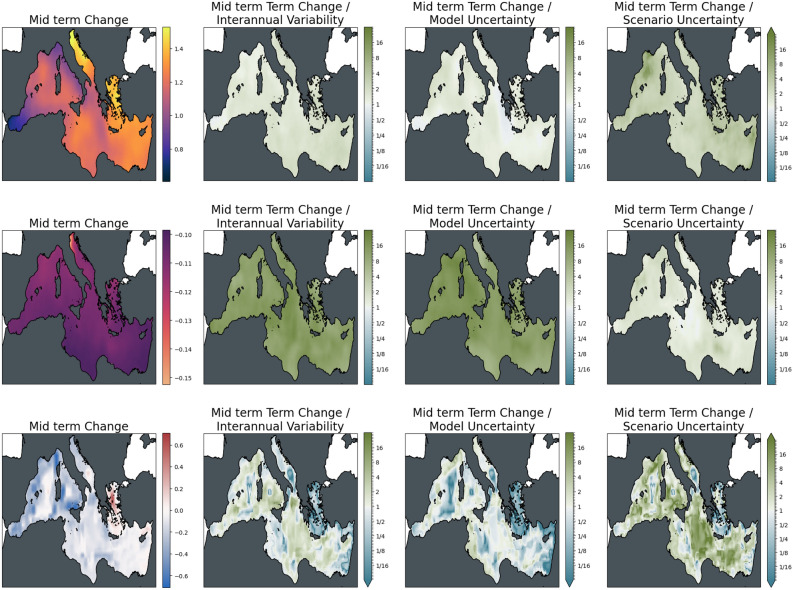
The magnitude of mid-term changes in the Mediterranean Sea under SSP2-4.5, against three sources of uncertainty for three ecosystem indicators. From left to right: changes between mid-term conditions (2041–2060 mean) and present-day conditions (1995–2014); changes relative to internal variability; changes relative to model uncertainty; changes relative to scenario uncertainty. Top to bottom: Surface Temperature (K); surface pH; bottom dissolved oxygen (ml/l).

**Figure 6:**
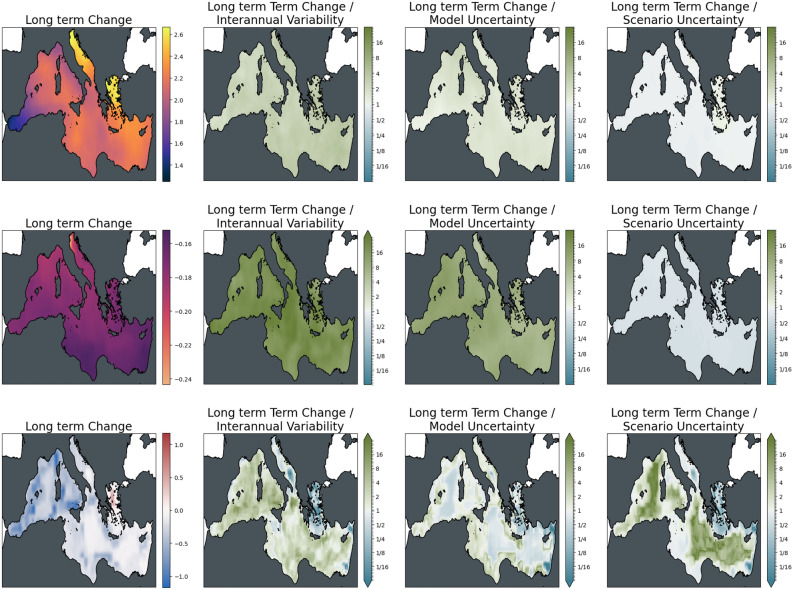
Strength of long-term climate-driven changes in the Mediterranean Sea under SSP2-4.5, against three sources of uncertainty for three ecosystem variables. From left to right: changes between long-term conditions (2081–2100 mean) and present-day conditions (1995–2014); changes relative to internal variability; changes relative to model uncertainty; changes relative to scenario uncertainty. Top to bottom: Surface Temperature (K); surface pH; bottom dissolved oxygen (ml/l).

To give a clearer picture of the relative importance of the different sources of uncertainty and their role in different locations, Figs. 5 and 6 show maps of the changes of the three indicators for the ensemble average of scenario SSP2-4.5 as absolute values and relative to the uncertainties. The increase in surface temperature is strongest in the Adriatic and Aegean Seas, with higher changes in the Eastern compared to the Western Basin of the Mediterranean Sea. Warming almost doubles from mid to the end of the century with no major difference in the spatial distribution of change. Mid-century interannual variability and model uncertainty are of the order of the changes across the basin, while the differences between the scenarios are significantly lower than the changes induced. This situation reverses for long-term changes, which become more significant with respect to interannual variability. At the same time, the difference between the scenarios has become larger in relative terms and is comparable to the magnitude of the change.

Acidification in the Mediterranean Sea is strongest in the Northern Adriatic, with a generally slightly higher decrease in pH in the Northern parts compared to the Southern coast of the basin. As can be inferred from the basin mean time series, changes are strongly significant for both time slices with respect to interannual variability and model uncertainty. At the same time, the difference between the mitigation pathways is on the order of the changes at mid-century and is much larger towards the end of the century.

For bottom dissolved oxygen, the situation is much less clear. While, on average, a decrease in seafloor oxygen is visible from the time series, some areas show an increase in oxygen for the ensemble mean (most evident in the Aegean Sea); interannual variability and, particularly, model uncertainty is high in these areas. Areas of oxygen decrease, on the contrary, emerge from interannual variability with changes slightly higher than the model uncertainty, although with some regional exceptions. Similarly, changes in scenario play a much more important role in areas of oxygen increase compared to areas of decrease. In the latter areas, differences among the scenarios were relatively small compared to the magnitude of the induced decrease in oxygen.

#### North Sea

**Figure 7:**
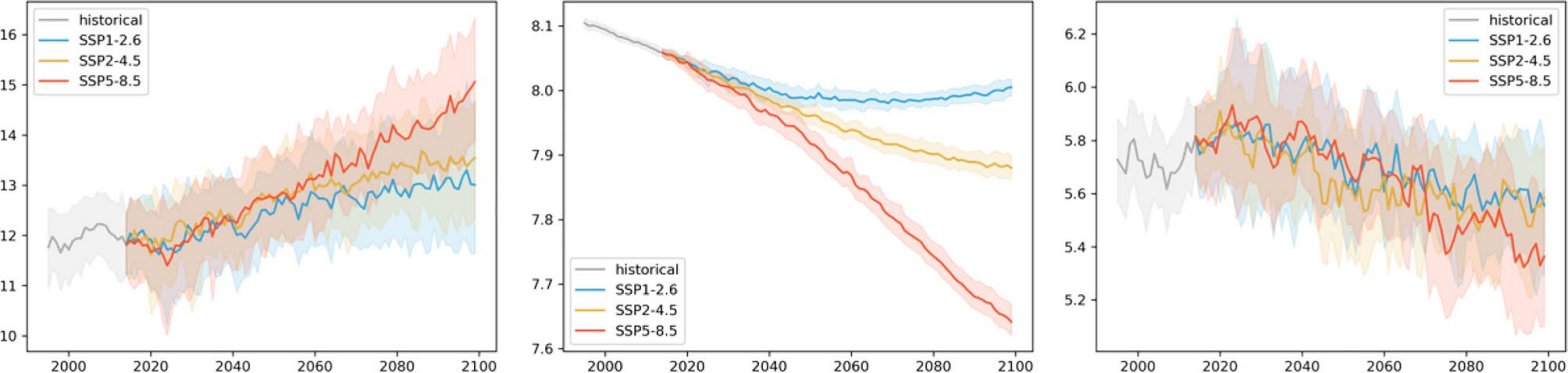
Time series of North Sea average surface temperature (°C), pH, and bottom oxygen (ml/l) over the historical time period and the three scenarios. Solid lines show the ensemble, and shaded areas show the ensemble spread based on the 2.5 and 97.5 percentiles of the model distributions.

The basin-scale mean evolution of greenhouse gas-induced changes in physical and biogeochemical pressures of the wider North Sea area (Fig. 7) is comparable to the Mediterranean Sea. Warming is, however, less accentuated in the North Sea compared to the Mediterranean Sea, with only a 3.0 °C increase projected at the end of the century for the no-mitigation scenario SSP5-8.5 and < 1.0 °C for the moderate and strong mitigation scenarios. Acidification ranges from slightly less than 0.1 units (SSP1-2.6) to around 0.5 units of decrease in surface pH (SPP5-8.5), while seafloor oxygen decrease by ~ 0.1–0.3 ml/l) with interannual variability up to ~ 0.2 ml/l).

**Figure 8:**
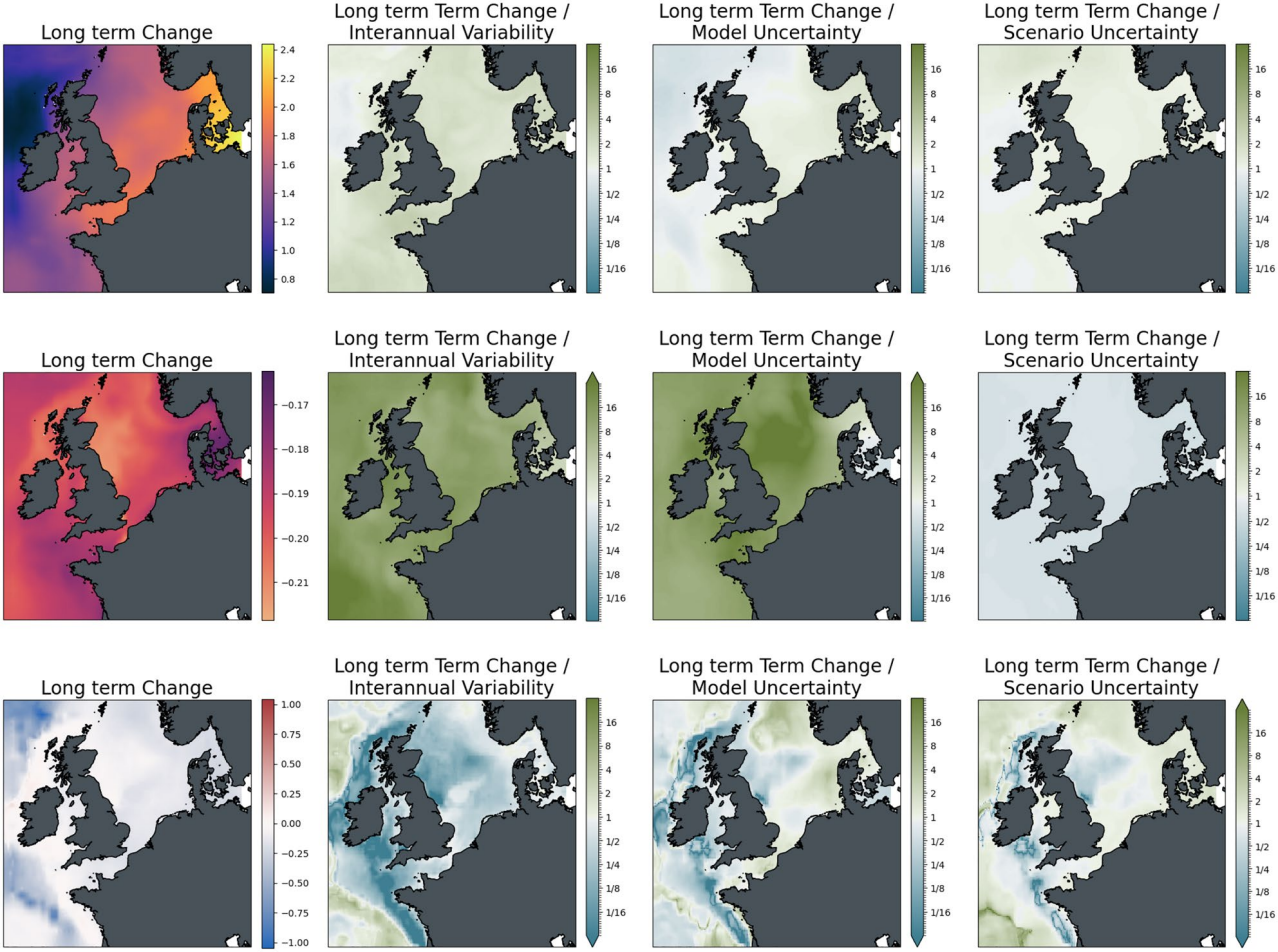
Significance of mid-term changes in the North Sea under SSP2-4.5, against three sources of uncertainty for three ecosystem indicators. From left to right: changes between mid-term conditions (2041–2060 mean) and present-day conditions (1995–2014); changes relative to internal variability; changes relative to model uncertainty; changes relative to scenario uncertainty. Top to bottom: Surface Temperature (K); surface pH; bottom dissolved oxygen (ml/l).

**Figure 9:**
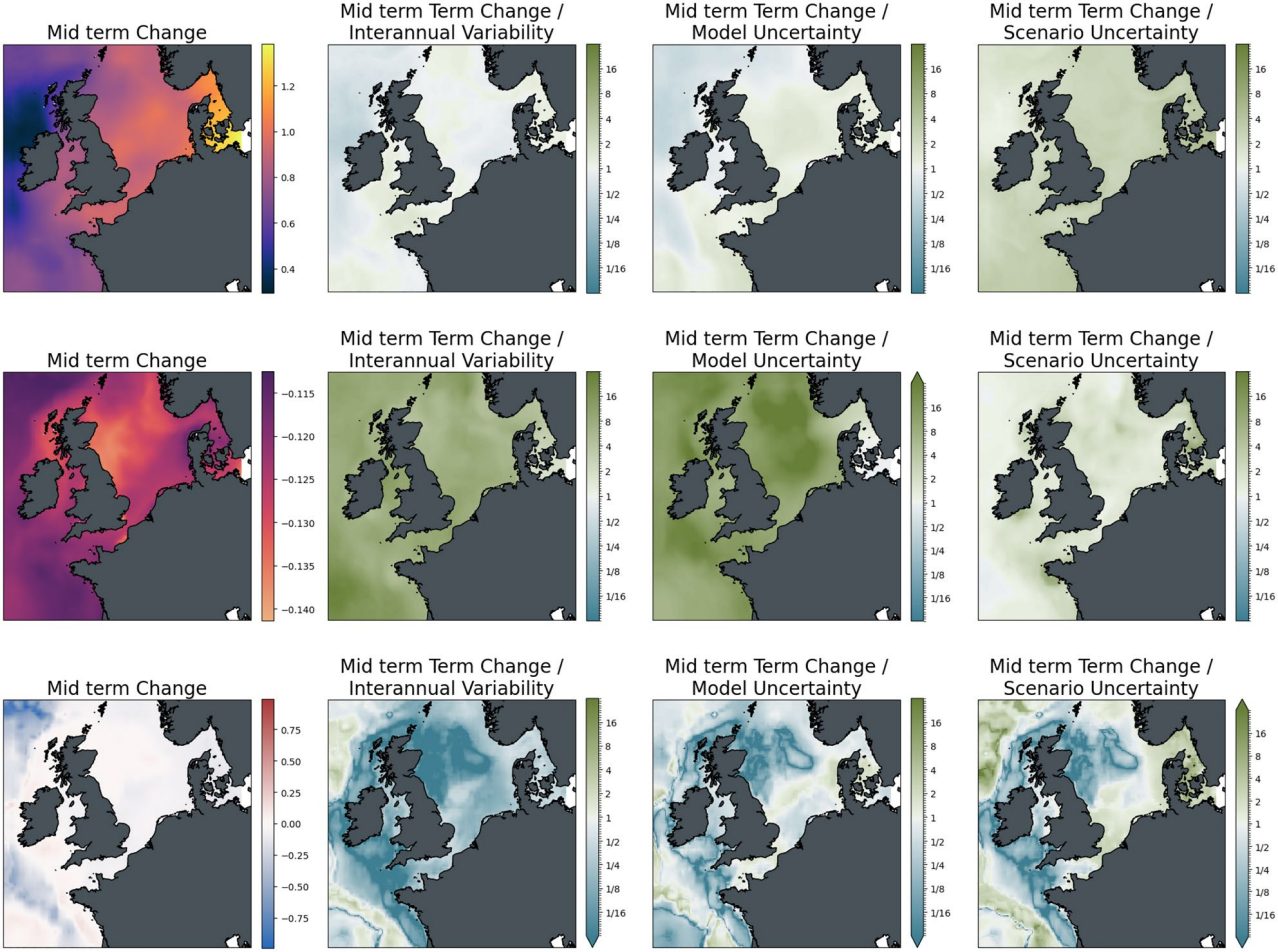
Significance of long-term changes in the North Sea under SSP2-4.5, against three sources of uncertainty for three ecosystem indicators. From left to right: changes between long-term conditions (2081–2100 mean) and present-day conditions (1995–2014); changes relative to internal variability; changes relative to model uncertainty; changes relative to scenario uncertainty. Top to bottom: Surface Temperature (K); surface pH; bottom dissolved oxygen (ml/l).

Considering the spatial distribution of changes and uncertainties (Figs. 8, 9) warming is strongest towards the Eastern parts of the European shelf and comparatively weak towards the open Atlantic Ocean. On most of the continental shelf, however, these trends are comparatively weak with respect to interannual variability and model uncertainty (ratio is only slightly > 1). The differences between the scenario pathways are only of minor importance at mid-century (approximately 1/3 of the change signal across the basin) but eventually reach about the same order of magnitude as those induced by long-term changes. Seafloor oxygen in the ensemble average changes noticeably only in the open ocean areas along the shelf break, where dissolved oxygen declines by up to 1 ml/l. These changes begin to emerge at mid-century but only become significant towards the end of the century. The difference in changes between greenhouse gas scenarios is minor, even towards the end of the century.

#### Bay of Biscay

**Figure 10:**
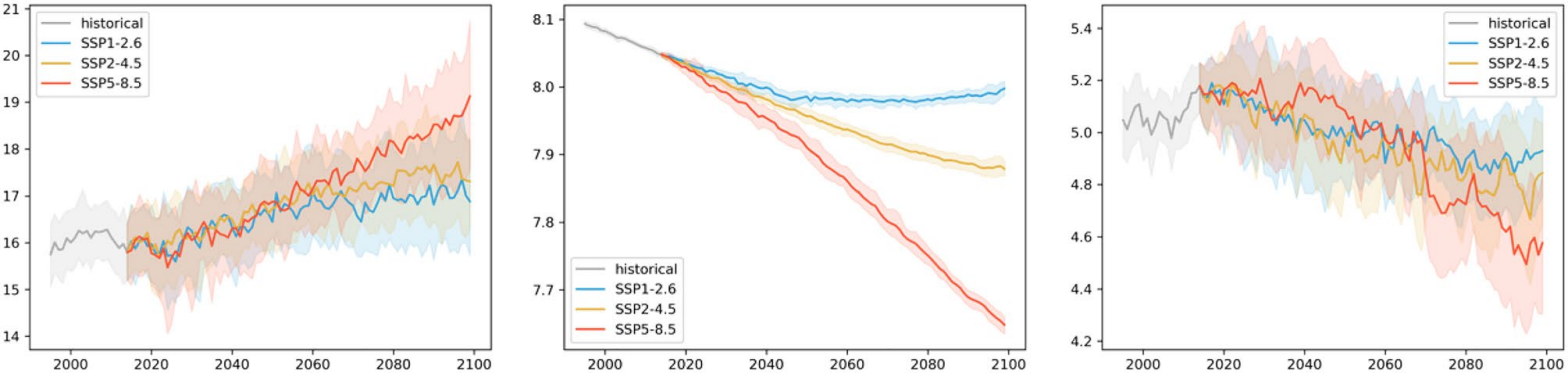
Time series of the Bay of Biscay average surface temperature (°C), pH, and bottom oxygen (ml/l) over the historical time period and the three scenarios. Solid lines show the ensemble, and shaded areas show the ensemble spread based on the 2.5 and 97.5 percentiles of the model distributions.

In the area around the Bay of Biscay, the domain averages roughly followed the patterns observed in the two previous regions with strong continuous warming up to 3.0 °C by 2100 and acidification of 0.4 pH units for SSP5-8.5 (Fig. 10). These changes are attenuated in the other two (moderate to strong mitigation) scenarios. For example, pH does not decrease but increases somewhat in the second half of the century for SSP1-2.6. Acidification trends were strongly significant, while the warming trends emerged less clearly due to considerable model uncertainty (~ 1.5° to 3.5°), particularly for the two pathways producing weaker changes. Deoxygenation shows considerable model (~ 0.4–0.5 ml/l) and interannual (up to 0.2 ml/l) variability. The trend in deoxygenation are minimal or absent for the strongest (SSP5-8.5) scenario through 2040, followed by a few years of strong interannual variability and a rapid decline that continues through the end of this century. There is a weaker, more continuous deoxygenation in the other two scenarios, similar to patterns in the North and Mediterranean Seas. The unexpected observed climate variability across scenarios could be indicative of infrequent and quasi-stochastic regime shifts which are an expected component of natural variability^34^. Still, filtering out the inter-annual variability suggests that the trend is consistent across the scenarios.

**Figure 11:**
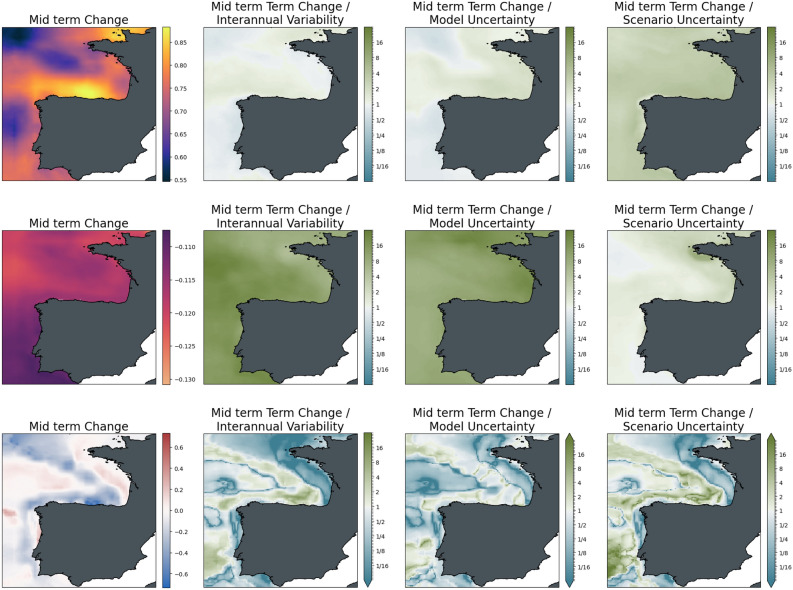
Significance of mid-term changes in the Bay of Biscay under SSP2-4.5, against three sources of uncertainty for three ecosystem indicators. From left to right: changes between mid-term conditions (2041–2060 mean) and present-day conditions (1995–2014); changes relative to internal variability; changes relative to model uncertainty; changes relative to scenario uncertainty. Top to bottom: Surface Temperature (K); surface pH; bottom dissolved oxygen (ml/l).

**Figure 12:**
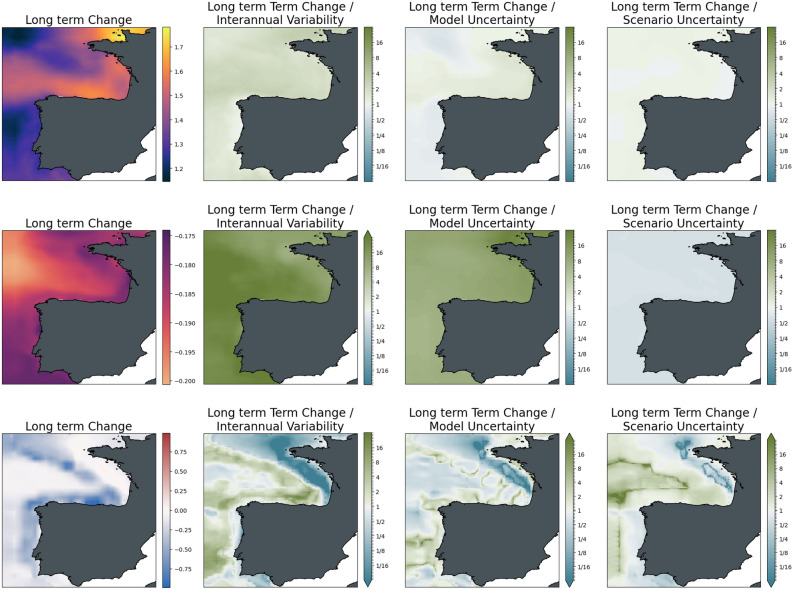
Significance of long-term changes in the Bay of Biscay under SSP2-4.5, against three sources of uncertainty for three ecosystem indicators. From left to right: changes between long-term conditions (2081–2100 mean) and present-day conditions (1995–2014); changes relative to internal variability; changes relative to model uncertainty; changes relative to scenario uncertainty. Top to bottom: Surface Temperature (K); surface pH; bottom dissolved oxygen (ml/l).

The spatial distribution of these average patterns in the domain (Figs. 11, 12) indicated the Northern coast of the Iberian Peninsula and the northern coast of Brittany as hotspots of surface warming, with the former particularly strong in the mid-term (up to 1°) and the latter particularly strong in the long-term (almost 2°). Relative to interannual variability and model uncertainty, however, this warming is only slightly emergent in the mid-term, while both interannual and model uncertainties are high in the deeper Atlantic waters. In the long-term, changes become significant with respect to interannual variability, while model uncertainty remains persistent through the end of the century. Consistent with patterns in the other regional seas, differences in scenario pathways in warming are small in the mid-term, while in the long-term, the different mitigation strategies will lead to differences in warming of the same order of magnitude as the change itself. Oxygen is expected to increase on the Celtic and Armorican shelfs although the three sources of uncertainty remain high for both mid and long term. While in the deeper Atlantic waters and the coastal waters of northern Spain and the western coast of Portugal oxygen will decrease, and changes are significant with respect to interannual variability. For model and scenario uncertainty changes in oxygen are pre-dominantly significant with exceptions such as the inner coastal domain of Portugal which is dominated by upwelling and more complex oceanographic processes. Acidification trends are comparatively homogeneous across the domain, with somewhat stronger trends in the off-shelf areas of the North-Eastern Atlantic. This pattern is consistent between the two time slices; however, acidification is about 50% higher at the end of the century compared to the mid-century. The trends are strongly significant with respect to model uncertainty and interannual variability for both time slices. The difference between scenarios is of the same order of magnitude as the induced changes at mid-century, while at the end of the century, the mitigation pathways become increasingly important as the difference between scenarios reaches twice the magnitude of the change signal.

#### Baltic Sea

**Figure 13:**
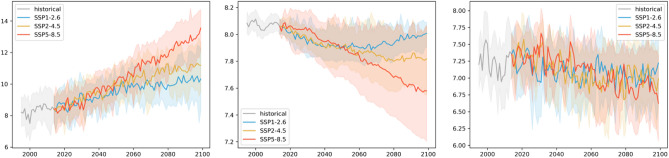
Time series of Baltic Sea average surface temperature (॰C), pH, and bottom oxygen (ml/l) over the historical time slice and the three scenarios. Full lines show the ensemble, and shaded areas show the ensemble spread based on the 2.5 and 97.5 percentiles of the model distribution.

While the basin mean warming, acidification, and deoxygenation trends are also present in the Baltic Sea, their behaviour and relation to uncertainty are substantially different in this coastal, semi-enclosed basin compared to the other regions. A fundamental difference is the large model uncertainty (up to 0.8 pH units) and increased interannual variability (up to 0.05 units) of surface pH with respect to the induced changes (0.1–0.5 pH units) (Fig. 13). In addition, the deoxygenation change (~ 0.2 ml/l) here is significantly weaker, and interannual variability is significantly higher (up to 0.5 ml/l). Warming trends (2–5 °C), by contrast, are comparable to the other basins.

**Figure 14:**
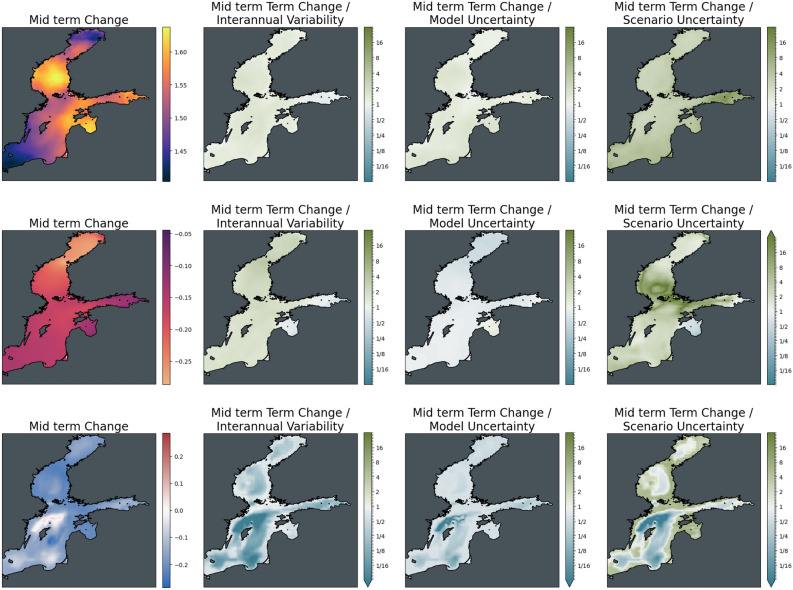
Significance of mid-term changes in the Baltic Sea under SSP2-4.5, against three sources of uncertainty for three ecosystem indicators. From left to right: changes between mid-term conditions (2041–2060 mean) and present-day conditions (1995–2014); changes relative to internal variability; changes relative to model uncertainty; changes relative to scenario uncertainty. Top to bottom: Surface Temperature (K); surface pH; bottom dissolved oxygen (ml/l).

**Figure 15:**
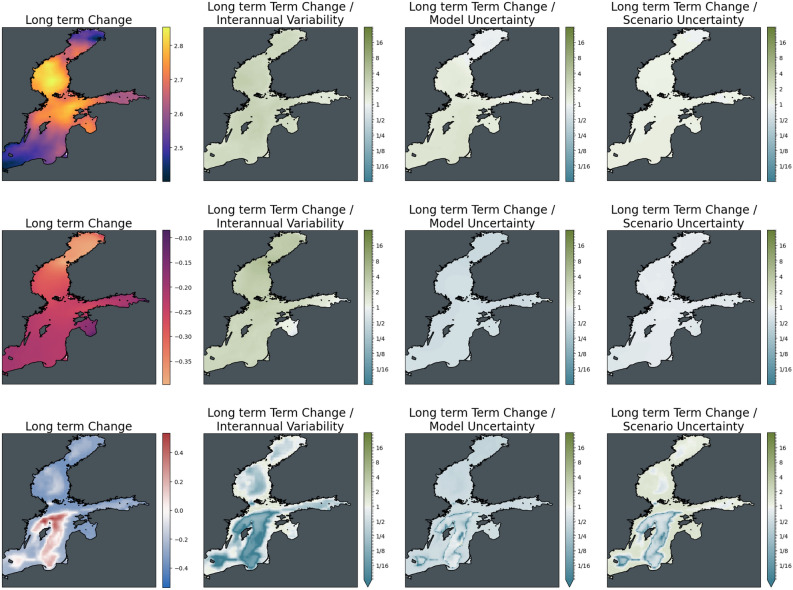
Significance of long-term changes in the Baltic Sea under SSP2-4.5, against three sources of uncertainty for three ecosystem indicators. From left to right: changes between long-term conditions (2081–2100 mean) and present-day conditions (1995–2014); changes relative to internal variability; changes relative to model uncertainty; changes relative to scenario uncertainty. Top to bottom: Surface Temperature (K); surface pH; bottom dissolved oxygen (ml/l).

Looking at regional differences in these trends (Figs. 14, 15), warming in the ensemble average of the Baltic Sea for scenario SSP2-4.5 at mid-century is strongest in the Bothnian Sea and the Gulf of Riga and weakest at the margins of the Bothnian Bay and the Southern Baltic Proper. This pattern also persists at the end of the century with the two warming hotspots spreading into the Northern Baltic Proper. Compared to interannual variability and model uncertainty, the trends only weakly emerge across the basin. Differences between mitigation pathways are negligible at mid-century but reach the order of magnitude of the induced changes by 2100. For acidification, which is strongest in the Bothnian Bay, there is a clear distinction in the impact of interannual variability and model uncertainty on the significance of the mid-and long-term trend. While trends clearly emerge from interannual variability, they are subject to model uncertainty, as was visible already in the basin average time series that reaches more than twice the level of the trend.

The bottom oxygen concentration at mid-century reveals deoxygenation across the whole basin except for a small region of increase in oxygen north of Gotland that extends to the whole Gotland Basin at the end of the century. It should be noted, however, that these changes are comparatively uncertain with respect to interannual variability and model uncertainty across the entire Baltic Sea and are particularly uncertain in oxygen increase. This area is also the only area in which scenario differences in oxygen trends are larger than the actual oxygen trend, making it an area of uncertain outcome in all aspects.

## Discussion

Many governments have made Nature-based Solutions ecosystem-based management, habitat restoration, and adaptive marine spatial planning a core part of their climate adaptation and planning^35^. However, such efforts demand accounting for impacts of costal biological and physical ocean dynamics and variability which further requires biophysical model results that are at a finer resolution than the CMIP outputs. Statistical downscaling can provide the necessary data to perform rapid analysis including estimates of the uncertainties involved while ensuring high skill when compared with historical observations. Our results suggest warming is evident across all regions, fully emerging from the background uncertainties related to internal variability and model differences in the second half of the century, with substantial differences between the mitigation pathways. Acidification significantly emerges from model uncertainty and internal variability in the historical climate, while the different climate mitigation scenarios lead to distinct trajectories in surface pH before mid-century. Although deoxygenation appears to be present across all domains, the signal is weaker compared to temperature and pH in terms of model uncertainty and internal variability, and the impact of different greenhouse gas trajectories is much less distinct. These qualitative characteristics vary considerably in extent and exhibit substantial local heterogeneity within each domain, underlining the importance of a spatially explicit and high-resolution approach to providing projections of the impacts of anthropogenic climate change on marine ecosystem components, functions, and services.

Statistically downscaled ensemble products, such as the one presented here, can be useful for calculating exposure terms for climate risk assessments performed across large areas, such as that conducted for threatened species within marine protected areas throughout the Mediterranean^36^ or for fisheries across European regional seas^37^. Ongoing studies along the European coastline are utilizing downscaled climate data to better understand how preservation of natural habitat-forming species such as mangroves, seagrasses, kelp forests, or coral reefs can buffer impacts from storms, and sea-level rise, as well as contribute as carbon sinks. In some cases, first-hand knowledge of the oceanography and marine biogeochemistry of an area is essential to decide whether an ensemble climate product is representative of an area. Here we use a model reanalysis, the GLORYS12V1 at 1/12th degree resolution, for the ocean physics and a model hindcast, the GOBH at ¼ degree resolution, for the biogeochemistry as a baseline for the bias-correction and downscaling, which particularly in the Baltic Sea is too coarse to resolve many of the local features. In addition, the performance of the downscaling will be limited by the skill of the hindcast GOBH model. Still, we argue that, for biogeochemical variables, model hindcasts are currently the only comprehensive datasets consistently available across the regions and vertical layers of this study, even though efforts to fill this gap are ongoing, e.g., via reanalysis products and extrapolation of observational datasets using artificial intelligence. We believe that the downscaled product nevertheless substantially improves upon the original CMIP6 models for the same region. Future improvements can be made if an enhanced biological hindcast, reanalysis or observational products are developed at sufficient resolution and time coverage.

Generally, the utilization of ocean biogeochemical models for understanding the fluctuations and transformations in marine environments, arising from both natural and human-induced influences, has surged in recent decades. The growth in the use of these tools can be attributed to the emergence of computers capable of executing trillions of calculations within seconds, resulting in global high-resolution ocean reanalyses such as GLORYS12V1, providing researchers detailed information on the marine environment. Still, to perform global future projections at high spatial resolution, the cost and time required is considerable. In fact, an increase in horizontal resolution by a factor of two increases the computational cost by ten^38^. Instead, we rely on global coarse resolution models that provide less detail but are faster to run, which can be further downscaled locally to hold a proper resolution useful for projecting coastal processes. Most often, these downscaled models are dynamic, meaning that they calculate the full set of hydrodynamic equations for a limited domain and use the coarse-resolution global models as boundary forcing. Using dynamical models for simulating one model domain is time-consuming and expensive. As a result, dynamical models are often constrained to downscale a few, or a small subset, of global climate models and scenarios, which limits their flexibility and hampers an assessment of sources of uncertainty. Alternatively, the statistical downscaling described in this study, provides a more rapid way of assessing local coastal impacts of climate change for a subset of relevant variables. While both dynamic and statistical approaches have their advantages and disadvantages, they are complementary. For example, one can apply a statistical downscaling approach to effectively gain an understanding of expected coastal climate impacts across a range of scenarios and climate models as has been done here for European regional seas. These findings can inform the selection of locations that require a more refined dynamical model for a comprehensive assessment of the three-dimensional effects of climate change on local ecosystems. This knowledge facilitates the optimal utilization of models and strategic application to enhance scientific efforts in understanding and addressing future ecological impacts.

Statistical downscaling has shortcomings that must be recognized when using the ensemble data. First, for the dataset presented here, we limit downscaling to individual depth levels. This limits analysis to two dimensions instead of three, which, as one example, is acceptable if you are analysing shellfish distributions under changing environmental conditions^39^, but inadequate if you need to understand the vertical distribution of a diel vertical migrator like krill. The DQM methodology also assumes that the biases at quantiles are stationary in time i.e., the functional relationship between the observed values and the GCM/ESM for the historical time period holds for the future^9^. This assumption can cause problems with extreme values as their historical distribution is expected to continue in the future when we know that climate change can lead to novel non-linear states^40^. In a recent paper the DQM approach was a favoured methodology as it preserved the climate change signal and trend^41^ compared to other methods like traditional Quantile Mapping (QM^9^) where the mean of the raw climate change signal (CCS) is not preserved but altered to correct for biases in the GCM/ESM. Choosing DQM suggests that you have confidence in the GCM/ESM circulation pattern and skill, as the resulting downscaling will maintain the inherent CCS^9^. Challenges with statistical downscaling, such as inflating or deflating extreme values during downscaling, and considerations as what should be regarded as best practice is an ongoing process where new developments and approaches are published frequently^42–44^. In addition, dynamical downscaling becomes fundamental when dynamic consistency across multiple variables is required in the downstream applications, such as subsequent modelling studies that require the coherent representation of dynamic features that may be lost across variables applying statistical approaches.

Regional downscaling provides detailed local climate projections for researchers, stakeholders, and management entities to understand, mitigate, and adapt to climate change. Choosing between dynamical and statistical downscaling depends on the application and research question(s). A dynamical model is effective for understanding a region's structure and dynamics under climate change but is subject to bias^8^ and lacks the breadth and coordinated protocols of global experiments. These characteristics hinder the ability to conduct a thorough evaluation of sources for proper uncertainty assessment, thereby limiting confidence in the projections. Statistical downscaling is a good alternative, particularly when applied to individual variables or depth levels, but lacks dynamic consistency and probably will not predict unobserved phenomena (e.g., synergistic, or antagonistic multivariate processes). Despite its widespread use in atmospheric science^42,43,45^, statistical downscaling is rarely applied to ocean models^46^. In fact, to our knowledge, this is the first ocean downscaling that uses a detrended quantile mapping approach to downscale both physical and biogeochemical ocean properties. This downscaling provided climate projections at a regional scale across all European waters across a range of climate models and scenarios at reasonable computational cost.

### Supplementary Information

Supplementary Information.

## Data Availability

The ensemble projections datasets covering European waters for three scenarios, SSP1-2.6, SSP2-4.5, and SSP5-8.5, are available on Zenodo DOI: 10.5281/zenodo.6523925^47^ and from individual DOIs for each region: (1) North Sea: 10.5281/zenodo.6523926 (2) Mediterranean Sea: 10.5281/zenodo.6523899 (3) Baltic Sea: 10.5281/zenodo.6524111 (4) Bay of Biscay: 10.5281/zenodo.6524142.
