# Supplementary material for: Statistically downscaled CMIP6 ocean variables for European waters
Source: Sci Rep. 2024 Jan 12;14:1209. doi: 10.1038/s41598-024-51160-1 (PMC10786869; doi:10.1038/s41598-024-51160-1)
Supplement: Supplementary file 1 — Supplementary Information. [file 41598_2024_51160_MOESM1_ESM.docx]

**Supplementary information for “Statistically downscaled CMIP6 ocean variables for European waters”**

Trond Kristiansen^1,4,5^, Momme Butenschön^2^, and Myron Peck^3^

^1^Farallon Institute, Petaluma, California, USA

^2^Ocean and Data Assimilation Divicion, Fondazione Centro Euro-Mediterraneo sui Cambiamenti Climatici, Bologna, Italy

^3^Department of Coastal Systems, Royal Netherlands Institute for Sea Research, Texel, the Netherlands

^4^Actea Inc, San Francisco, California, USA

^5^Norwegian Institute for Water Research, Oslo, Norway

*Correspondence to*: Trond Kristiansen ([trondkr@faralloninstitute.org](mailto:trondkr@faralloninstitute.org))

| **variable** | **CMCC-CM2-SR5** | **CMCC-ESM2** | **GFDL-ESM4** | **IPSL-CM6A-LR-r1** | **IPSL-CM6A-LR-r3** | **MPI-ESM1-2-LR** | **ensemble** |
| --- | --- | --- | --- | --- | --- | --- | --- |
| **MED surface thetao** | 0.980 | 0.980 | 0.980 | 0.980 | 0.980 | 0.980 | 0.980 |
| **NorthSea surface thetao** | 0.985 | 0.985 | 0.985 | 0.985 | 0.985 | 0.985 | 0.985 |
| **BayOfBiscay surface thetao** | 0.988 | 0.988 | 0.988 | 0.989 | 0.989 | 0.988 | 0.989 |
| **BalticSea surface thetao** | 0.931 | 0.933 | 0.933 | 0.932 | 0.933 | 0.932 | 0.932 |
| **MED surface o2** |  | 0.932 |  | 0.932 | 0.931 | 0.931 | 0.932 |
| **NorthSea surface o2** |  | 0.924 |  | 0.928 | 0.928 | 0.929 | 0.927 |
| **BayOfBiscay surface o2** |  | 0.863 |  | 0.857 | 0.858 | 0.857 | 0.859 |
| **BalticSea surface o2** |  | 0.862 |  | 0.858 | 0.858 | 0.861 | 0.860 |

**Table S1: Pearson correlation of the individual and ensemble downscaled models for surface temperature (thetao) and surface oxygen (o2) for each basin. The evaluation is based on present-day seasonal averages against WOA climatology.**

| **variable** | **CMCC-CM2-SR5** | **CMCC-ESM2** | **GFDL-ESM4** | **IPSL-CM6A-LR-r1** | **IPSL-CM6A-LR-r3** | **MPI-ESM1-2-LR** | **ensemble** |
| --- | --- | --- | --- | --- | --- | --- | --- |
| **MED surface thetao** | 0.972 | 0.970 | 0.972 | 0.972 | 0.972 | 0.972 | 0.971 |
| **NorthSea surface thetao** | 0.978 | 0.978 | 0.977 | 0.978 | 0.978 | 0.978 | 0.978 |
| **BayOfBiscay surface thetao** | 0.981 | 0.982 | 0.981 | 0.981 | 0.981 | 0.981 | 0.981 |
| **BalticSea surface thetao** | 0.912 | 0.917 | 0.912 | 0.914 | 0.912 | 0.917 | 0.915 |
| **MED surface o2** |  | 0.829 |  | 0.835 | 0.836 | 0.835 | 0.833 |
| **NorthSea surface o2** |  | 0.847 |  | 0.854 | 0.854 | 0.856 | 0.852 |
| **BayOfBiscay surface o2** |  | 0.814 |  | 0.803 | 0.802 | 0.801 | 0.806 |
| **BalticSea surface o2** |  | 0.630 |  | 0.635 | 0.635 | 0.627 | 0.631 |

**Table S2: Spatial Kling-Gupta Efficiency of the individual and ensemble downscaled models for surface temperature (thetao) and surface oxygen (o2) for each basin. The evaluation is based on present-day seasonal averages against WOA climatology.**

| **variable** | **CMCC-CM2-SR5** | **CMCC-ESM2** | **GFDL-ESM4** | **IPSL-CM6A-LR-r1** | **IPSL-CM6A-LR-r3** | **MPI-ESM1-2-LR** | **ensemble** |
| --- | --- | --- | --- | --- | --- | --- | --- |
| **MED surface thetao** | 0.201 | 0.202 | 0.199 | 0.200 | 0.199 | 0.200 | 0.200 |
| **NorthSea surface thetao** | 0.177 | 0.177 | 0.177 | 0.177 | 0.177 | 0.177 | 0.177 |
| **BayOfBiscay surface thetao** | 0.156 | 0.157 | 0.155 | 0.155 | 0.155 | 0.156 | 0.155 |
| **BalticSea surface thetao** | 0.474 | 0.472 | 0.472 | 0.475 | 0.470 | 0.472 | 0.473 |
| **MED surface o2** |  | 0.389 |  | 0.387 | 0.387 | 0.387 | 0.387 |
| **NorthSea surface o2** |  | 0.493 |  | 0.485 | 0.486 | 0.484 | 0.486 |
| **BayOfBiscay surface o2** |  | 0.736 |  | 0.761 | 0.763 | 0.764 | 0.753 |
| **BalticSea surface o2** |  | 1.182 |  | 1.255 | 1.249 | 1.178 | 1.189 |

**Table S3: Spatial Normalised Root-Mean-Square difference of the individual and ensemble downscaled models for surface temperature (thetao) and surface oxygen (o2) for each basin. The evaluation is based on present-day seasonal averages against WOA climatology.**

| **variable** | **CMCC-CM2-SR5** | **CMCC-ESM2** | **GFDL-ESM4** | **IPSL-CM6A-LR-r1** | **IPSL-CM6A-LR-r3** | **MPI-ESM1-2-LR** | **ensemble** |
| --- | --- | --- | --- | --- | --- | --- | --- |
| **MED surface thetao** | 0.981 | 0.978 | 0.981 | 0.981 | 0.982 | 0.982 | 0.981 |
| **NorthSea surface thetao** | 0.995 | 0.997 | 0.995 | 0.994 | 0.994 | 0.994 | 0.995 |
| **BayOfBiscay surface thetao** | 1.003 | 0.999 | 1.007 | 1.005 | 1.005 | 1.007 | 1.004 |
| **BalticSea surface thetao** | 0.975 | 0.978 | 0.992 | 0.981 | 0.982 | 0.977 | 0.980 |
| **MED surface o2** |  | 0.839 |  | 0.846 | 0.848 | 0.847 | 0.844 |
| **NorthSea surface o2** |  | 0.905 |  | 0.904 | 0.905 | 0.904 | 0.904 |
| **BayOfBiscay surface o2** |  | 1.026 |  | 1.045 | 1.047 | 1.051 | 1.040 |
| **BalticSea surface o2** |  | 1.087 |  | 1.088 | 1.088 | 1.085 | 1.087 |

**Table S4: Spatial Ratio of Standard Deviations (Model/Observations) of the individual and ensemble downscaled models for surface temperature (thetao) and surface oxygen (o2) for each basin. The evaluation is based on present-day seasonal averages against WOA climatology.**

| **variable** | **CMCC-CM2-SR5** | **CMCC-ESM2** | **GFDL-ESM4** | **IPSL-CM6A-LR-r1** | **IPSL-CM6A-LR-r3** | **MPI-ESM1-2-LR** | **ensemble** |
| --- | --- | --- | --- | --- | --- | --- | --- |
| **MED surface thetao** | 1.001 | 1.001 | 1.000 | 1.001 | 1.001 | 1.001 | 1.001 |
| **NorthSea surface thetao** | 1.000 | 1.000 | 1.000 | 1.000 | 1.000 | 1.000 | 1.000 |
| **BayOfBiscay surface thetao** | 1.000 | 1.000 | 1.000 | 1.000 | 1.000 | 1.000 | 1.000 |
| **BalticSea surface thetao** | 1.001 | 1.001 | 1.001 | 1.001 | 1.001 | 1.001 | 1.001 |
| **MED surface o2** |  | 0.995 |  | 0.995 | 0.995 | 0.996 | 0.995 |
| **NorthSea surface o2** |  | 0.998 |  | 1.001 | 1.001 | 1.001 | 1.000 |
| **BayOfBiscay surface o2** |  | 1.006 |  | 1.008 | 1.008 | 1.008 | 1.007 |
| **BalticSea surface o2** |  | 0.990 |  | 0.989 | 0.989 | 0.990 | 0.989 |

**Table S5: Spatial Ratio of Means (Model/Observations) of the individual and ensemble downscaled models for surface temperature (thetao) and surface oxygen (o2) for each basin. The evaluation is based on present-day seasonal averages against WOA climatology.**

| **variable** | **CMCC-CM2-SR5** | **CMCC-ESM2** | **GFDL-ESM4** | **IPSL-CM6A-LR-r1** | **IPSL-CM6A-LR-r3** | **MPI-ESM1-2-LR** | **ensemble** |
| --- | --- | --- | --- | --- | --- | --- | --- |
| **MED surface thetao** | 0.802 | 0.829 | 0.793 | 0.802 | 0.803 | 0.823 | 0.840 |
| **NorthSea surface thetao** | 0.778 | 0.798 | 0.746 | 0.691 | 0.723 | 0.719 | 0.789 |
| **BayOfBiscay surface thetao** | 0.891 | 0.908 | 0.920 | 0.862 | 0.861 | 0.910 | 0.924 |
| **BalticSea surface thetao** | 0.796 | 0.837 | 0.842 | 0.904 | 0.906 | 0.916 | 0.911 |
| **MED surface o2** |  | 0.903 | 0.893 | 0.877 | 0.871 | 0.816 | 0.906 |
| **NorthSea surface o2** |  | 0.796 | 0.764 | 0.654 | 0.675 | 0.683 | 0.698 |
| **BayOfBiscay surface o2** |  | 0.889 | 0.836 | 0.834 | 0.834 | 0.818 | 0.893 |
| **BalticSea surface o2** |  | 0.734 | 0.691 | 0.871 | 0.871 | 0.798 | 0.852 |

**Table S6: Spatial Pearson Correlation of the original and ensemble Earth System Models for surface temperature (thetao) and surface oxygen (o2) for each basin. The evaluation is based on present-day seasonal averages against WOA climatology.**

| **variable** | **CMCC-CM2-SR5** | **CMCC-ESM2** | **GFDL-ESM4** | **IPSL-CM6A-LR-r1** | **IPSL-CM6A-LR-r3** | **MPI-ESM1-2-LR** | **ensemble** |
| --- | --- | --- | --- | --- | --- | --- | --- |
| **MED surface thetao** | 0.687 | 0.697 | 0.690 | 0.677 | 0.667 | 0.691 | 0.683 |
| **NorthSea surface thetao** | 0.724 | 0.734 | 0.657 | 0.621 | 0.662 | 0.616 | 0.718 |
| **BayOfBiscay surface thetao** | 0.860 | 0.881 | 0.768 | 0.702 | 0.704 | 0.864 | 0.832 |
| **BalticSea surface thetao** | 0.628 | 0.665 | 0.760 | 0.883 | 0.896 | 0.782 | 0.820 |
| **MED surface o2** |  | 0.638 | 0.725 | 0.654 | 0.627 | 0.535 | 0.609 |
| **NorthSea surface o2** |  | 0.675 | 0.544 | 0.559 | 0.579 | 0.535 | 0.525 |
| **BayOfBiscay surface o2** |  | 0.818 | 0.669 | 0.651 | 0.652 | 0.764 | 0.739 |
| **BalticSea surface o2** |  | 0.079 | 0.394 | 0.629 | 0.629 | 0.531 | 0.556 |

**Table S7: Spatial Kling-Gupta Efficiency of the original and ensemble Earth System Models for surface temperature (thetao) and surface oxygen (o2) for each basin. The evaluation is based on present-day seasonal averages against WOA climatology.**

| **variable** | **CMCC-CM2-SR5** | **CMCC-ESM2** | **GFDL-ESM4** | **IPSL-CM6A-LR-r1** | **IPSL-CM6A-LR-r3** | **MPI-ESM1-2-LR** | **ensemble** |
| --- | --- | --- | --- | --- | --- | --- | --- |
| **MED surface thetao** | 2.365 | 2.273 | 2.357 | 2.612 | 2.656 | 2.430 | 2.364 |
| **NorthSea surface thetao** | 1.549 | 1.428 | 1.646 | 1.759 | 1.733 | 1.540 | 1.492 |
| **BayOfBiscay surface thetao** | 1.339 | 1.272 | 1.488 | 1.653 | 1.687 | 1.515 | 1.349 |
| **BalticSea surface thetao** | 4.637 | 4.222 | 4.376 | 4.874 | 4.809 | 4.153 | 4.239 |
| **MED surface o2** |  | 1.093 | 1.174 | 1.310 | 1.344 | 1.270 | 1.827 |
| **NorthSea surface o2** |  | 0.990 | 1.331 | 1.299 | 1.318 | 1.242 | 2.983 |
| **BayOfBiscay surface o2** |  | 0.943 | 1.229 | 1.517 | 1.568 | 1.470 | 4.148 |
| **BalticSea surface o2** |  | 4.304 | 5.272 | 4.547 | 4.547 | 5.402 | 6.866 |

**Table S8: Spatial Normalised Root-Mean-Square difference of the original and ensemble Earth System Models for surface temperature (thetao) and surface oxygen (o2) for each basin. The evaluation is based on present-day seasonal averages against WOA climatology.**

| **variable** | **CMCC-CM2-SR5** | **CMCC-ESM2** | **GFDL-ESM4** | **IPSL-CM6A-LR-r1** | **IPSL-CM6A-LR-r3** | **MPI-ESM1-2-LR** | **ensemble** |
| --- | --- | --- | --- | --- | --- | --- | --- |
| **MED surface thetao** | 0.844 | 0.833 | 0.900 | 0.829 | 0.823 | 0.832 | 0.788 |
| **NorthSea surface thetao** | 0.885 | 0.885 | 1.153 | 0.847 | 0.873 | 0.813 | 0.880 |
| **BayOfBiscay surface thetao** | 0.948 | 0.949 | 1.196 | 0.758 | 0.761 | 0.922 | 0.857 |
| **BalticSea surface thetao** | 1.182 | 1.212 | 1.070 | 1.029 | 1.000 | 1.009 | 0.943 |
| **MED surface o2** |  | 0.668 | 0.862 | 0.758 | 0.730 | 0.617 | 0.566 |
| **NorthSea surface o2** |  | 0.899 | 1.234 | 0.900 | 0.918 | 0.804 | 0.685 |
| **BayOfBiscay surface o2** |  | 0.981 | 1.181 | 0.721 | 0.723 | 0.950 | 0.715 |
| **BalticSea surface o2** |  | 1.783 | 1.126 | 0.982 | 0.982 | 1.018 | 0.881 |

**Table S9: Spatial Ratio of Standard Deviations (Model/Observations) of the original and ensemble Earth System Models for surface temperature (thetao) and surface oxygen (o2) for each basin. The evaluation is based on present-day seasonal averages against WOA climatology.**

| **variable** | **CMCC-CM2-SR5** | **CMCC-ESM2** | **GFDL-ESM4** | **IPSL-CM6A-LR-r1** | **IPSL-CM6A-LR-r3** | **MPI-ESM1-2-LR** | **ensemble** |
| --- | --- | --- | --- | --- | --- | --- | --- |
| **MED surface thetao** | 1.002 | 1.001 | 1.000 | 0.998 | 0.997 | 0.998 | 1.000 |
| **NorthSea surface thetao** | 1.003 | 1.001 | 1.000 | 1.001 | 1.001 | 1.000 | 1.001 |
| **BayOfBiscay surface thetao** | 1.001 | 1.000 | 1.002 | 0.997 | 0.997 | 0.997 | 0.998 |
| **BalticSea surface thetao** | 1.007 | 1.003 | 0.999 | 1.000 | 1.000 | 0.998 | 1.001 |
| **MED surface o2** |  | 0.991 | 1.022 | 1.030 | 1.032 | 1.025 | 0.848 |
| **NorthSea surface o2** |  | 0.994 | 1.011 | 1.001 | 1.005 | 1.005 | 0.833 |
| **BayOfBiscay surface o2** |  | 0.998 | 0.998 | 1.027 | 1.029 | 1.033 | 0.849 |
| **BalticSea surface o2** |  | 1.000 | 1.072 | 1.041 | 1.041 | 0.923 | 0.850 |

**Table S10: Spatial Ratio of Means (Model/Observations) of the original and ensemble Earth System Models for surface temperature (thetao) and surface oxygen (o2) for each basin. The evaluation is based on present-day seasonal averages against WOA climatology.**

**
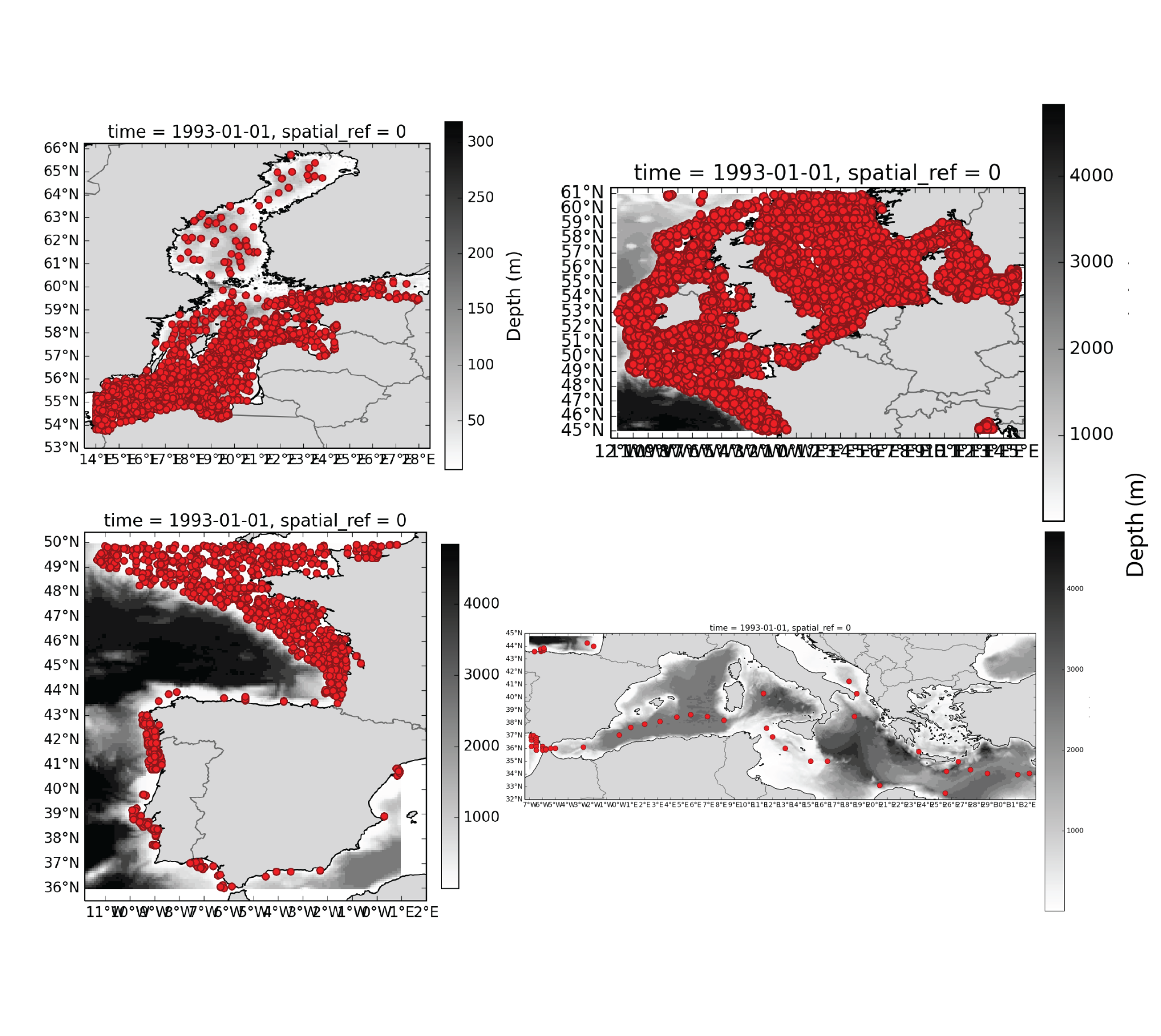
**

**Figure S1: Map of the locations where bottom oxygen was collected across regions of the Baltic Sea (upper left), the North Sea (upper right), the Bay of Biscay (lower left), and the Mediterranean (lower right). For the Mediterranean Sea we used the GLODAP database, while for the rest of the regions, we used the ICES database.**

**
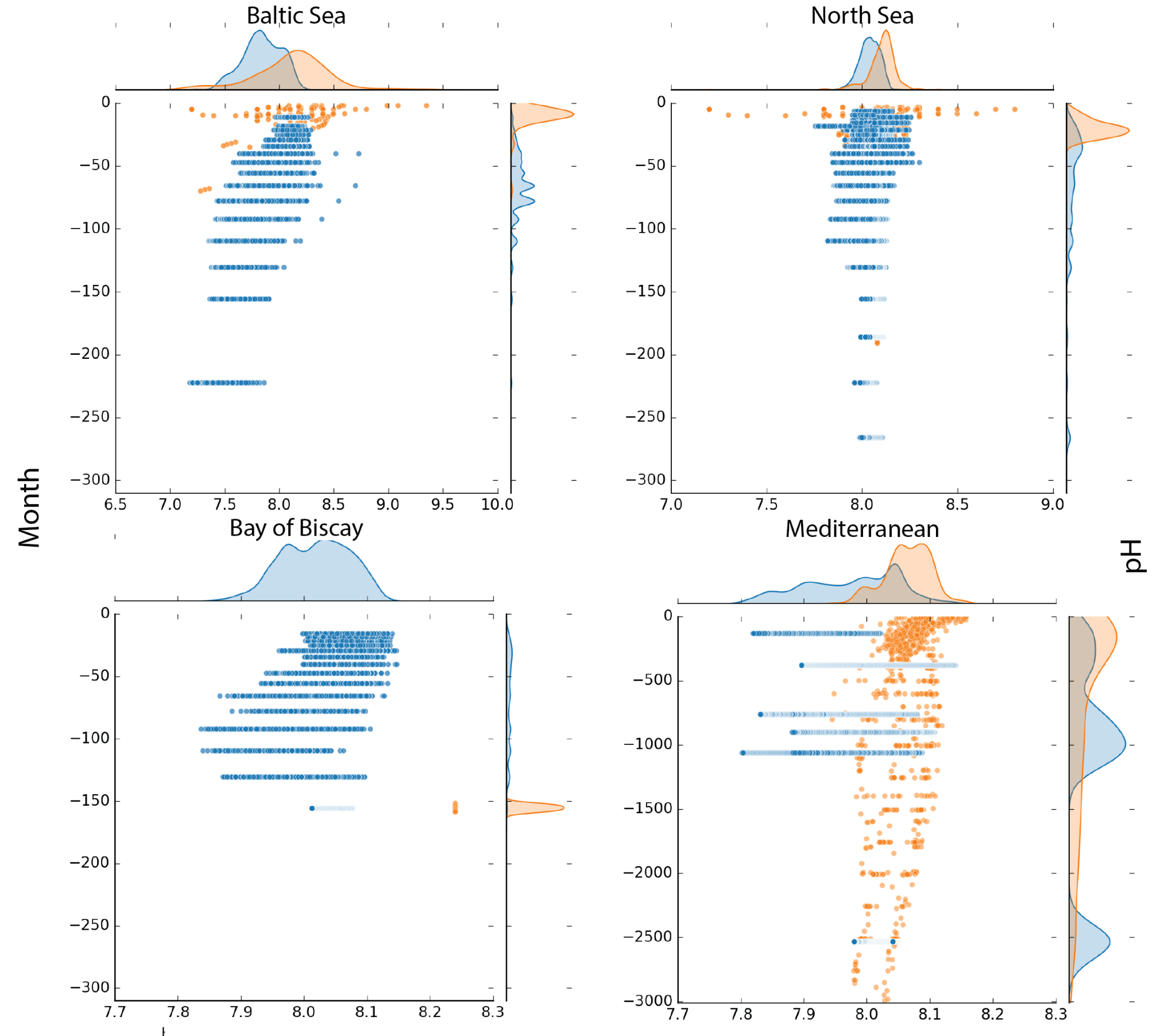
**

**Figure S2. Comparison between the ensemble (blue) downscaled bottom pH (ph) and shipboard observations (orange) extracted from the ICES (**[**www.ices.dk**](http://www.ices.dk)**) and the GLODAP databases** [**(Lauvset et al. 2022)**](https://paperpile.com/c/8y5Oyd/NTgK)**. For the Baltic Sea (upper left), the North Sea (upper right), and the Bay of Biscay (lower left), we used data from ICES for the comparison, while for the Mediterranean Sea, we used GLODAP. The comparison used all available data for the period 1993-2020. For pH, we had very few observed datapoints of pH that matched the criteria used to filter the datasets. pH is on total scale.**
